# Fecal carriage of ESBL and Carbapenemase-producing Enterobacteriaceae, and its associated factors among hospital and non-hospital janitors at the University of Gondar, Northwest Ethiopia: A comparative cross-sectional study

**DOI:** 10.1371/journal.pone.0355041

**Published:** 2026-07-31

**Authors:** Amanuale Zayede, Yitayih Wondimeneh, Sirak Biset, Mucheye Gizachew

**Affiliations:** 1 Department of Medical Microbiology, School of Biomedical and Laboratory Sciences, College of Medicine and Health Sciences, University of Gondar, Gondar, Ethiopia; 2 Department of Medical Laboratory Sciences, College of Health Science, Woldia University, Woldia, Ethiopia; Universidad San Francisco de Quito, ECUADOR

## Abstract

**Background:**

Extended-spectrum beta-lactamase (ESBL) and carbapenemase-producing Enterobacteriaceae (CPE) pose a critical public health threat. Janitors are routinely exposed to contaminated environments and may act as a reservoir for these resistant bacteria, particularly in resource-limited settings like Ethiopia, where data on this subject are scarce.

**Objective:**

The aim of this study was to assess fecal carriage rates of ESBL-PE, CPE and its associated factors among hospital and non-hospital janitors at the University of Gondar (UoG), Northwest Ethiopia.

**Methods:**

A comparative cross-sectional study was conducted from June 15 to September 25, 2025, at the UoG. A total of 385 janitors (203 hospital and 182 non-hospital) were selected using stratified and simple random sampling techniques. Data on socio-demographic and associated factors were collected using a semi-structured questionnaire. Stool samples were collected aseptically and cultured on MacConkey agar. Antimicrobial resistance (AMR) patterns were determined by the Kirby-Bauer disk diffusion method following Clinical and Laboratory Standards Institute (CLSI) 2024 guidelines. The ESBL and carbapenemase production were confirmed using the combination disk and modified Carbapenem inactivation methods, respectively. Quality control was ensured by using standard reference strains. Data analysis was performed using SPSS version 27.0, employing chi-square tests and logistic regression. A *p*-value of <0.05 was considered statistically significant.

**Result:**

Among the 385 stool samples, a total of 416 Enterobacteriaceae isolates were recovered, 229 from hospital janitors and 187 from non-hospital janitors. The overall fecal carriage rates among janitors were 83 (21.6%) for ESBL-PE and 14 (3.6%) for CPE. Hospital janitors had significantly higher carriage rate than non-hospital janitors for both ESBL-PE (28.6% vs. 13.7%, *p* < 0.001) and CPE (5.9% vs. 1.1%, *p* = 0.012), respectively. *Escherichia coli* was the predominant ESBL-producer. Significant risk factors identified for ESBL-PE colonization included lack of hand hygiene at home, history of antibiotic use, and urinary tract infection for hospital janitors, and age ≥ 40 years and history of non-prescription antibiotic use were for non-hospital janitors.

**Conclusion and recommendations:**

This high carriage rates of ESBL-PE and CPE, identify janitors as a significant reservoir for resistant bacteria. We recommend integrating janitors into national AMR surveillance programs and strengthening infection prevention and control and antimicrobial stewardship training.

## Introduction

Enterobacteriaceae are a large family of Gram-negative bacteria (GNB) that includes pathogens in the genus *Escherichia*, *Klebsiella*, *Salmonella*, *Shigella*, and *Enterobacter*. These bacteria can cause a wide range of infections in the skin and soft tissue, bloodstream, urinary, respiratory, cardiovascular, and central nervous systems, both in healthcare settings and the community [1,2]. Of particular concern are Carbapenem and third-generation cephalosporin-resistant Enterobacteriaceae*,* which the World Health Organization (WHO) has classified them as critical-priority pathogens due to their high resistance to last-resort antibiotics [3].

A major mechanism of resistance in these bacteria is the production of beta-lactamases, enzymes that hydrolyze and inactivate beta-lactam antibiotics. The extended-spectrum beta-lactamases (ESBLs) are a particularly dangerous subset of these enzymes that can break down third-generation cephalosporins and monobactams [4,5]. Resistance to these antibiotics is acquired by plasmid-mediated mutation, either by amino acid substitution in the active site such as the case for Temonieara (TEM) or sulfhydryl reagent variable (SHV; class A) enzymes or by inter bacteria gene transfer in case of Cefotaxime-Munich (CTX-M) [6,7]. Based on the Ambler molecular classification, ESBLs primarily belong to Class A and Class D serine beta-lactamases. Class A includes common plasmid-mediated enzymes such as TEM, SHV, and CTX-M, which are inhibited by clavulanate. However, class D consists of the oxacillinases (OXA)-type enzymes, often characterized by their resistance to clavulanate and strong activity against oxacillin [8]. In the Bush-Jacoby functional classification, ESBLs are categorized under Group 2be, which includes serine beta-lactamases that are inhibited by clavulanic acid. Furthermore, some OXA-type ESBLs are also classified under Group 2d, which includes clavulanate-resistant enzymes [9].

Additionally, Enterobacteriaceae also produce carbapenemase, enzymes that hydrolyze and inactivate Carbapenem antibiotics [10]. Based on the Ambler molecular classification, carbapenemase are found across three classes: Class A *K. pneumoniae* carbapenemase (KPC), Class B Metallo-beta-lactamases (MBLs), including; New Delhi Metallo-beta-lactamase (NDM), Verona Integron-encoded Metallo-beta-lactamase (VIM), Imipenemase (IMP), and Class D OXA-type enzymes. Class A and D enzymes use an active-site serine for hydrolysis, while Class B relies on zinc ions [8]. In the Bush-Jacoby functional classification, carbapenemases are grouped primarily in Group 2f (serine carbapenemases), Group 3 (Metallo-beta-lactamases), and Group 2d (some OXA-type enzymes with carbapenemase activity) [9].

The ESBL and carbapenemase-producing Enterobacteriaceae (CPE) are among the most urgent antimicrobial resistance (AMR) threats, as identified by the Centers for Disease Control and Prevention (CDC) and WHO [3,11]. These pathogens severely limit treatment options, as ESBL-PE often require Carbapenem, while CPE infections may only respond to older, less effective antibiotics such as aminoglycosides, polymyxins, and fosfomycin [12]. The spread of these resistant bacteria leads to higher mortality rates, prolonged hospital stays, and increased healthcare costs [13,14].

Experts predict that by 2050, AMR could cause approximately 1.91 million deaths annually worldwide. Without effective interventions, cumulative AMR-related deaths may reach 39.1 million between 2025 and 2050, demanding urgent global action to mitigate this crisis [15]. The 2022 WHO survey found that only 15.2% of healthcare facilities met all minimum infection prevention and control (IPC) requirements, contributing to the high burden of HAIs [16]. *Escherichia coli* and *K. pneumoniae* are two common species of Enterobacteriaceae that possess pathogenic capabilities and carry ESBL-encoding genes [5]. While IPC training and education are commonly directed towards health care staff, the WHO also recommends extending these efforts to all staff working in health care facilities, including cleaning and housekeeping personnel [17].

Recent studies from Ethiopia highlight a significant local burden of antimicrobial-resistant bacteria. A 2023−24 community study in Addis Ababa found ESBL-producing Enterobacteriaceae (ESBL-PE) in 31.4% of healthy individuals, with carbapenemase-producers (CPE) at 0.8%; isolates were predominantly *E. coli* (96.3%) [18]. An institutional based study conducted among food handlers in Dilla, 2019.Showed that, 25.3% of isolates were ESBL-PE, mostly *E. coli* (78.4%) [19]. Another similar institution-based study also conducted in 2021 among food handlers at the UoG cafeterias, Gondar. The study reported that 21.7% and 2.4% of stool sample were confirmed as ESBL-PE and CPE, respectively, with the most prevalent ESBL-PE was *E. coli* (14.8%), followed by *K. pneumoniae* (5.9%) [20].

The transmission of ESBL-PE and CPE occurs through multiple reservoirs, including colonized patients, contaminated medical equipment, hospital sinks, drains, sewage systems, and even food products [21,22]. Additionally, the gut microbiota can serve as a reservoir for resistance genes, facilitating their transfer to pathogenic strains [23].

Janitors (also called cleaners or custodians) play a vital role in reducing infections by maintaining hygiene and disinfecting contaminated surfaces as they are responsible for removing biohazardous waste, including human excreta and radioactive materials [24]. However, despite their critical role, they are often low-wage, and low-status workers who are exposed to physical, chemical, biological, and psychosocial hazards [25,26]. Therefore, this study was conducted with an aim to assess fecal carriage of ESBL-PE, CPE, and its associated factors among hospital and non-hospital janitors at the University of Gondar (UoG).

## Materials and methods

### Study setting study design and period, and setting

A comparative institution-based cross-sectional study was conducted from June 15 to September 25, 2025, among hospital and non-hospital janitors at the UoG. The UoG is one of the oldest and most prominent higher education institutions in Northwest Ethiopia. It is situated in Gondar city, which is part of the Central Gondar Zone in the Amhara National Regional State. It is approximately 738 kilometers Northwest of Addis Ababa, the capital of Ethiopia, and about 180 kilometers from Bahir Dar, the capital of the Amhara National Regional State. The coordinates of Gondar city are 12°36′ N latitude and 37°28′ E longitude, with an elevation of 2,133 meters above sea level. Gondar city is renowned as one of Ethiopia’s ancient and populous cities, currently hosting two public referral hospitals, one private general hospital, and eight governmental health centers [27].

University of Gondar (UoG) comprises five campuses: the College of Medicine and Health Sciences (CMHS), Maraki, Atse Tewodros, Atse Fasile, and Tseda [28]. The CMHS includes the University of Gondar comprehensive specialized hospital (UoGCSH), which serves more than 13 million people in the catchment area, and has 28 hospital wards and 15 different outpatient service areas, with 960 beds. In 2024, approximately 400,000 clients visited outpatient clinics and more than 30,000 admission cases are seen annually. The hospital serves as a referral center in Northwest Ethiopia, as well as a teaching and research center. The Department of Medical Microbiology at the UoG includes a central bacteriology laboratory, which specializes in bacterial culture for diagnosis, research, and teaching [29]. According to the Human Resource Directorate of the UoG, there are currently 710 Janitors at the UoG, of whom 400 are working in the hospital area.

### Population

#### Source population.

All hospital and non-hospital janitors working at the UoG were the source population.

#### Study population.

All hospital and non-hospital janitors working at the UoG during the data collection period were the study population.

### Eligibility criteria

#### Inclusion criteria.

Hospital and non-hospital janitors at the UoG who have been employed for at least three months prior to data collection were included.

#### Exclusion criteria.

**Hospital janitors:** Janitors employed by the UoGCSH and are working in its administration building, cafeteria and gardens were excluded. In addition, janitors who have taken antibiotics within the past one week before the start of data collection were excluded.

**Non-hospital janitors:** Janitors employed by the UoG and have a history of working in hospital settings within the last six months prior to data collection were excluded [30,31]. In addition, janitors who have taken antibiotics within the past one week before the start of data collection were excluded.

### Variables

#### Dependent variables.

- Fecal carriage of ESBL-PE, and CPE.
- Antimicrobial resistance patterns of ESBL-PE, and CPE were dependent variables.

#### Independent variables.

Sociodemographic variables: Age, sex, educational status, marital status, family size, living with children.

Occupation related factors: Working area, department, work shift, service years, training regarding to IPC, access to appropriate PPE, access to handwashing facilities.

Hygiene related factors: Use gloves while cleaning, exposure to contaminated bodily fluids during cleaning procedures, hand washing habit at work place, methods used for hand cleaning, access to clean and safe toilet facilities at home, practice hand hygiene at home, fingernail status, source of water for drinking.

Behavior related factors: Use of antibiotics without prescription, drinking unpasteurized milk, eating raw meat, eating raw vegetables.

Clinical related factors: History of antibiotics use, hospital admission, urinary tract infection, diarrhea, close contact with a hospitalized person in the last three months, and having chronic disease were independent variables [20,32,33].

### Sample size determination and sampling technique

#### Sample size determination.

The sample size was calculated using the formula for comparing two independent proportions [34]. A prevalence rate of 50% was assumed for both groups, as no previous studies have been conducted in Ethiopia regarding to fecal carriage of ESBL-PE and CPE among hospital and non-hospital janitors.

The minimum sample size is calculated as follows.


$$\begin{array}{c} n=\;\frac{{\left(\text{Z}\alpha {/}_{\text{2}}\;+\;{\text{Z}}_{\beta }\right)}^{\text{2}}\;*\;(({\text{P}}_{\text{1}}\;(\text{1}-\;{\text{P}}_{\text{1}})\;+\;{\text{P}}_{\text{2}}\;(\text{1}-\;{\text{P}}_{\text{2}}))}{({\text{P}}_{\text{1}}-\;{\text{P}}_{\text{2}}{)}^{\text{2}}} \end{array}$$

Were,

n = required sample size for each group

α = Type I error (level of significant)

β = Type Ⅱ error (1- β = power of the study)

Zα/_2_ = Z-value corresponding to the confidence level (for 95% confidence, Zα/_2_ = 1.96)

Z_β_ = Z-value corresponding to the power (for 80% power, and Z_β_ = 0.84)

Since, there is no previous data, based on assumption set P₁ - P_2_ = 10% (a small detectable difference). P₁ = 50%, P_2_ = 40% (10% difference)


$$\begin{array}{c} n=\;\frac{\left(\text{1}.\text{96 }+\;\text{0}.\text{84}{)}^{\text{2}}\;*\;((\text{0}.\text{5}\;(\text{1}-\;\text{0}.\text{5})\;+\;\text{0}.\text{4}\;(\text{1}-\;\text{0}.\text{4})\right)}{(\text{0}.\text{1}{)}^{\text{2}}}\;\approx \text{ 385 per group} \end{array}$$

A finite population correction was applied separately for each group due to the small total populations (hospital janitors, N = 400; non-hospital janitors, N = 310). The corrected sample size for each group was calculated using the formula: n *adjusted* = n/ [1 + (n – 1)/N]

For hospital janitors: n *adjusted=* 385**/** [1 + (385- 1)/400] ≈197

For non-hospital janitors: n *adjusted=* 385**/** [1 + (385- 1)/310] ≈172

Considering a 10% non-response rate (n adjusted = n initial/1 − non-response rate), the final minimum sample size is set at 411, with 219 hospital and 192 non-hospital janitors.

#### Sampling technique.

**Fig 1:**
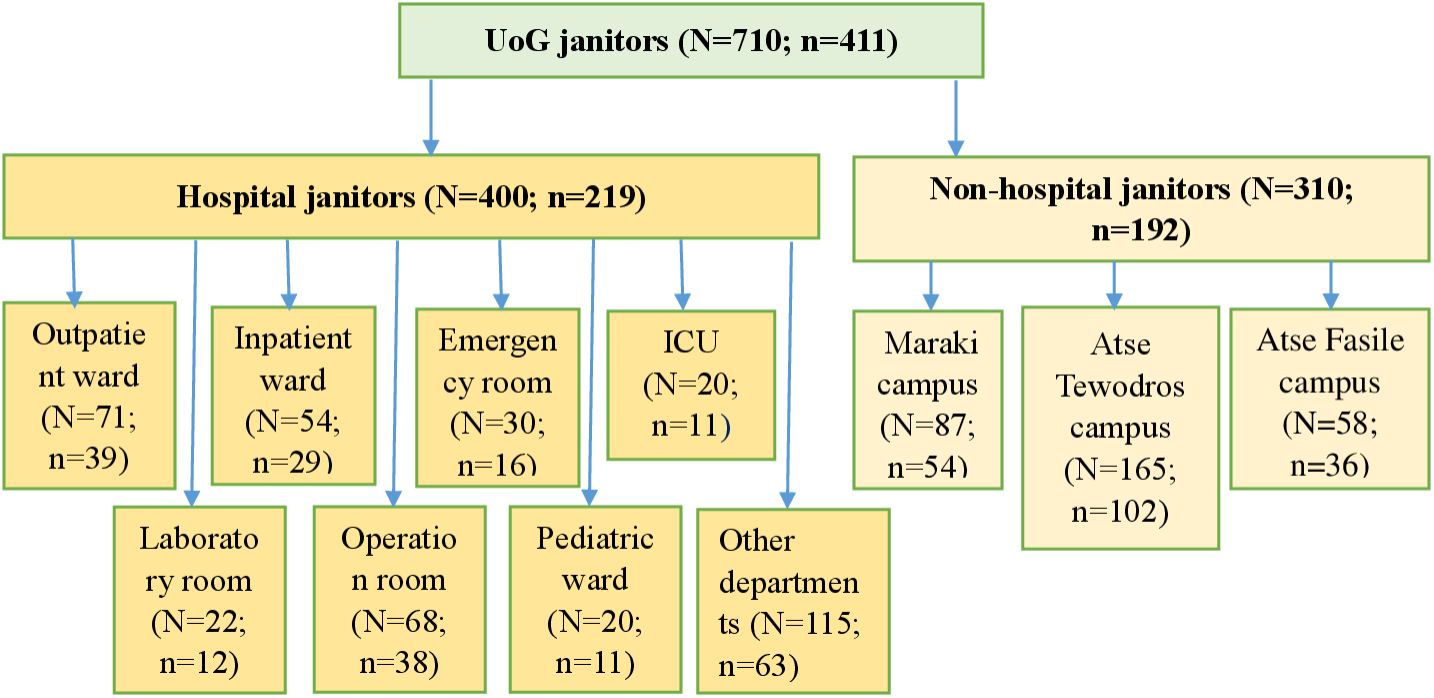
Flow of study participant selection using stratified and simple random sampling technique.

A stratified sampling technique combined with simple random sampling was used to select study participants (Fig 1).

A complete list of all janitors was obtained from the UoG Human Resource Directorate. This list was then divided into two strata: hospital and non-hospital janitors. The total sample size was proportionally allocated to each stratum based on the total number of janitors present in each of the stratum. Subsequently, participants were selected within each stratum using a simple random sampling technique, specifically the lottery method. This involved writing the names or identification numbers of all eligible janitors in a stratum on slips of paper, placing them in a container, and drawing them at random until the pre-determined sample size for that group was reached.

### Data collection and laboratory methods

#### Data collection tool and procedure.

Socio-demographic characteristics, hygiene practices, and behavioral, occupational, and clinical factors related to ESBL-PE and CPE were collected from study participants through face-to-face interviews using a semi- structured questionnaire (S1 File).

Following the interview, participants were instructed to collect a fresh stool sample of approximately 2 grams. The detailed procedure was as follows: first, they placed clean newspaper over the toilet seat opening. After passing stool onto the newspaper, they used a wooden stick to transfer a pea-sized portion preferably from any bloody, slimy, or watery areas into a clean, leak-proof container, ensuring no external spillage. The container lid was then tightly sealed. To prevent contamination by urine, participants were asked to empty their bladders prior to collection. Additionally, they were required to wash their hands with soap and water both before and after sample collection.

For stool samples that could not be cultured within 2 hours of collection, a sterile swab was inserted into the stool, rotated, and then placed into Cary-Blair medium (HI MEDIA Laboratories Pvt. Ltd., Mumbai, India), with the cap securely tightened. Each sample was then labeled with the sample number, date, and time of collection before placed in a cold box. All samples were transported to the School of Biomedical and Laboratory Sciences (SBMLS), Medical Microbiology Laboratory for examination [35,36]. Two trained laboratory technologists collected all data.

#### Laboratory methods.

**Enterobacteriaceae isolation and identification.** Stool samples collected from each participant were inoculated onto MacConkey agar (Oxoid Ltd, Basingstoke, United Kingdom (UK)), which were incubated aerobically at 37°C for 18–24 hours. Following incubation, all plates were examined for bacterial growth. Subsequently, sub-culturing on fresh MacConkey agar (Oxoid Ltd, Basingstoke, UK) was performed as necessary to obtain isolated, pure colonies for further characterization [37]. The purified isolates were then identified based on a combination of Gram staining (showing Gram-negative rods), colony morphology, and biochemical test results [38]. A series of biochemical tests (all from Oxoid Ltd, Basingstoke, UK) were included: Triple Sugar Iron agar to assess glucose/lactose/sucrose fermentation, gas production, and H_2_S production, lysine decarboxylase activity, indole production, citrate utilization, urea hydrolysis, and motility test (S2 File) [39].

**Antimicrobial susceptibility testing of isolates.** Antimicrobial susceptibility test (AST) was performed using the Kirby-Bauer disk diffusion method. The inoculum suspension was prepared in 0.85% sterile normal saline and then standardized to a 0.5 McFarland turbidity standard by visual comparison under adequate lighting against a white background with black contrasting lines. Mueller Hinton agar (MHA, Oxoid Ltd, Basingstoke, UK) plates were inoculated using the lawn culture method. The following antimicrobial disks (all from Oxoid Ltd, Basingstoke, UK) were applied to the MHA plates, which were incubated aerobically at 35°C ± 2°C for 16–18 hours: Amoxicillin-Clavulanic acid (20/10 µg), cefotaxime (30 μg), ceftazidime (30 μg), ceftriaxone (30 μg), cefoxitin (30 µg), meropenem (10 μg), imipenem (10 μg), gentamicin (10 μg), Amikacin (30 μg), Tetracycline (30 μg), Chloramphenicol (30 μg), Ciprofloxacin (5 μg), and trimethoprim-sulfamethoxazole (1.25/23.75 μg) [40,41]. After incubation, the diameters of the inhibition zones (including the disk diameter) were measured in millimeters (mm) using a calibrated ruler. The measured values were then interpreted as susceptible, intermediate, or resistant according to the Clinical and Laboratory Standards Institute (CLSI) 2024 guidelines (S1 Table) [40]. The multidrug-resistance (MDR) patterns of the isolates were identified using the criteria established by Magiorakos et al., 2012 [42].

**Identification and confirmation of ESBL-producing isolates.** All isolates were tested for susceptibility to ceftriaxone, cefotaxime, and ceftazidime using the Kirby-Bauer disk diffusion method to screen for potential ESBL-producers. Isolates with a zone of inhibition of ≤22 mm for Ceftazidime, ≤ 25 mm for Ceftriaxone, or ≤27 mm for Cefotaxime were considered potential ESBL-producers [40].

Phenotypic confirmation of ESBL-production was conducted using the combination disk test, following the CLSI 2024 guideline [40]. Pure colonies of the potential ESBL-PE isolates were emulsified in0.85% sterile normal saline to achieve a turbidity equivalent to a 0.5 McFarland standard. This suspension was then inoculated onto a MHA plate using the lawn culture method. The following antibiotic disks were applied, and then incubated aerobically at 35°C ± 2°C for 16–18 hours as recommended by CLSI 2024 guideline: Cefotaxime (30 µg), cefotaxime/clavulanic acid (30 µg/10 µg), ceftazidime (30 µg), and ceftazidime/clavulanic acid (30 µg/10 µg). An isolate was confirmed as an ESBL-PE if an increase of ≥5 mm in the zone diameter was observed for either antimicrobial agent tested in combination with Clavulanic acid compared to its zone diameter when tested alone [40].

**Identification and confirmation of carbapenemase-producing isolates.** Carbapenemase-producing Enterobacteriaceae (CPE) were screened using meropenem (10 µg) disks. A bacterial suspension was inoculated onto a MHA plate, and a Meropenem disk (10 µg) was placed on the agar surface. The plates were incubated at 35°C ± 2°C for 18–24 hours. An isolate with a zone of inhibition of ≤19 mm was regarded as a presumptive CPE [40].

Presumptive CPE isolates were confirmed using the modified Carbapenem inactivation method (mCIM). Briefly, a 1- µL loopful of the bacterial isolate was emulsified in 2 mL of tryptic soy broth (TSB), and a meropenem disk (10 µg) was immersed in the suspension. This was incubated for 4 hours ± 15 minutes at 35°C ± 2°C. Immediately following completion of the TSB-Meropenem disk suspension incubation, a suspension of the meropenem-susceptible *E. coli* control strain (ATCC 25922) was prepared in 0.85% sterile normal saline and adjusted to a 0.5 McFarland turbidity standard. This suspension was then inoculated onto MHA plate. The meropenem disk was transferred from the TSB to the inoculated MHA plate. This plate was then incubated at 35°C ± 2°C for 18–24 hours. A positive result for carbapenemase production was indicated by a zone of inhibition of 6–15 mm or the presence of a zone measuring 16–18 mm with pinpoint colonies within it [40].

### Data quality measures

The questionnaire was originally prepared in English, and then translated into local language (Amharic) for data collection, which was then translated back into English by an independent translator for analysis and reporting. A pre-test was conducted on 5% of the calculated sample size among janitors at the UoG, CMHS campus to assess validity and clarity. Close supervision was maintained by the principal investigator (PI) throughout the data collection period.

All laboratory procedures of sample collection, handling, storage, and transport were performed according to standard operating procedures (SOPs) and manufacturer recommendations. The sterility of newly prepared culture media was assessed by randomly selecting 5% of each batch, incubating them at 35°C ± 2°C for 16–18 hours, and confirming the absence of microbial growth. The performance of newly prepared culture media was checked using *E. coli* ATCC 25922, *E. faecalis* ATCC 29212, and *S. typhimurium* ATCC 14028 [43,44]. Additionally, the quality of antimicrobial disks was checked using *E. coli* ATCC 25922 (S2 Table) [40]. Furthermore, the viability of the 15% glycerol-TSB medium was tested by confirming the growth of *E. coli* ATCC 25922 both before and after storage. For the ESBL confirmatory tests, *K. pneumoniae* ATCC 700603 (positive control) and *E. coli* ATCC 25922 (negative control) were used. For carbapenemase detection, *K. pneumoniae* ATCC BAA (Bioresource Accession Number) 1705 and *K. pneumoniae* ATCC BAA-1706 were served as positive and negative controls, respectively. All confirmed Enterobacteriaceae isolates were stored at −20°C in 15% glycerol-TSB [40].

### Data management and analysis

All data were entered and managed using EpiData EntryClient and Manager, version 4.6.0.2 (EpiData Association, Odense, Denmark). Data were then exported to Statistical Package for the Social Sciences (SPSS), version 27.0 (IBM Corp., Armonk, NY, USA) for statistical analysis.

Descriptive statistics (frequencies and percentages) were computed to summarize the characteristics of the study population. Chi-square tests were used to assess for statistically significant differences in ESBL-PE and CPE fecal carriage between the hospital and non-hospital janitors. Bivariable and multivariable logistic regression analyses were used to identify factors associated with ESBL-PE carriage. Analysis of factors associated with CPE carriage was not performed due to the small number of CPE-positive participants, which failed to meet the assumptions for logistic regression model. Variables with a *p*-value of ≤0.25 in the bivariable analysis were included in the multivariable analysis. The results of these associations are presented as Crude Odds Ratios (COR) and Adjusted Odds Ratios (AOR) with corresponding 95% confidence intervals (CI). A *p*-value of <0.05 was considered statistically significant.

### Operational definitions

**Multidrug-resistant (MDR):** Bacteria that have developed non-susceptibility to at least one agent in three or more antimicrobial categories [42].

**Hospital janitors:** Personnel who are responsible for maintaining hygiene and preventing infections in healthcare facilities, they might work across an entire hospital or specialize in one specific department, may work full-time or part-time. This group includes janitors who clean and disinfect patient rooms, operating theaters, corridors, and frequently touched surfaces [45].

**Non-hospital janitors:** Also known as building service workers or custodians, are personnel responsible for maintaining cleanliness and hygiene in various settings outside healthcare facilities, such as administrative offices, schools, and public spaces to ensure a safe and sanitary environment [46].

### Ethical consideration

Ethical approval for the study was obtained from the Ethical Review Committee of the SBMLS at the UoG, with reference number SBMLS/997/, date 05 June 2025, prior to the start of any research activities.

Participation in the study was voluntary. Written informed consent was obtained from each participant after a thorough explanation of the study’s purpose, procedures, risks, and benefits. For participants who were unable to read, the information sheet was read to them in the presence of a witness, and they provided a fingerprint or a mark, which was countersigned by the witness. No minors were involved in this study.

Janitors who were found to be positive for fecal carriage of target bacteria were referred to their respective staff medical centers for further diagnosis and appropriate health education on IPC. The confidentiality of all participants was maintained throughout the study. All laboratory test results were used solely for the purposes of this research. Identifying information, such as names, was not collected on the data collection forms (S1 File).

## Results

### Sociodemographic characteristics of study participants

The initial calculated sample size was 411. With a response rate of 93.7%, the study was finally conducted among 385 participants (203 hospital janitors and 182 non-hospital janitors). The predominant study participants were females 373 (96.9%), aged between 20 and 51 years. The mean age was higher among hospital janitors (31.5 ± 5.99 years), compared to non-hospital janitors (29.1 ± 5.5 years). Over half of the participants, 215 (55.8%), held a diploma or higher degree, and 216 (56.1%) had more than five years of work experience. The majority were married 239 (62.1%), lived with their children 236 (61.3%), and had a family size of less than five people, 368 (95.6%) (Table 1).

**Table 1:** Sociodemographic characteristics of hospital (N = 203) and non-hospital janitors (N = 182) at the UoG, Northwest Ethiopia, June to September 2025.

Variables	Categories	Study Participants		Total (N = 385); n (%)
		Hospital Janitors (N = 203); n (%)	Non-hospital Janitors (N = 182); n (%)	
Gender	Male	10 (4.9)	2 (1.1)	12 (3.1)
	Female	193 (95.1)	180 (98.9)	373 (96.9)
Age	18-28	75 (36.9)	117 (64.3)	192 (49.9)
	29-39	113 (55.7)	56 (30.8)	169 (43.9)
	≥40	15 (7.4)	9 (4.9)	24 (6.2)
Level of Education	No formal education	3 (1.5)	4 (2.2)	7 (1.8)
	Primary school	13 (6.4)	8 (4.4)	21 (5.5)
	Secondary school	73 (35.9)	69 (37.9)	142 (36.9)
	Diploma and above	114 (56.2)	101 (55.5)	215 (55.8)
Marital Status	Single	57 (28.1)	58 (31.9)	115 (29.9)
	Married	122 (60.1)	117 (64.3)	239 (62.1)
	Divorced	18 (8.9)	7 (3.8)	25 (6.5)
	Widowed	6 (2.9)	0	6 (1.5)
Family size	1-5	188 (92.6)	180 (98.9)	368 (95.6)
	>5	15 (7.4)	2 (1.1)	17 (4.4)
Living with children’s	Yes	134 (66.0)	102 (56.0)	236 (61.3)
	No	69 (34.0)	80 (44.0)	149 (38.7)
Service years	1-5	79 (38.9)	90 (49.5)	169 (43.9)
	>5	124 (61.1)	92 (50.5)	216 (56.1)

### Occupational and hygiene related characteristics of study participants

A higher proportion of hospital janitors reported receiving IPC training, 124 (61.1%) compared to 28 (15.4%), non-hospital janitors. Additionally, a greater percentage of hospital janitors had non-trimmed fingernails, 66 (32.5%) compared to 33 (18.1%), non-hospital janitors (Table 2).

**Table 2:** Occupational and hygiene related characteristics of hospital (N = 203) and non-hospital janitors (N = 182) at the UoG, Northwest Ethiopia, June to September 2025.

Variables	Categories	Study Participants		Total (N = 385); n (%)
		Hospital Janitors (N = 203); n (%)	Non-hospital Janitors (N = 182); n (%)	
Receiving IPC training	Yes	124 (61.1)	28 (15.4)	152 (39.5)
	No	79 (38.9)	154 (84.6)	233 (60.5)
Access to PPE	Yes	157 (77.3)	79 (43.4)	236 (61.3)
	Sometimes	33 (16.3)	101 (55.5)	134 (34.8)
	No	13 (6.4)	2 (1.1)	15 (3.9)
Hand washing access	Always available	150 (73.9)	76 (41.8)	226 (58.7)
	Often unavailable	18 (8.9)	101 (55.5)	119 (30.9)
	No access	35 (17.2)	5 (2.7)	40 (10.4)
Use glove during cleaning	Always	197 (97.0)	174 (95.6)	371 (96.4)
	Only for dirty tasks	4 (2.0)	6 (3.3)	10 (2.6)
	Rarely	2 (1.0)	2 (1.1)	4 (1.0)
	Never	0	0	0
Exposure to contaminated surfaces	Frequently	20 (9.8)	73 (40.1)	93 (24.2)
	Occasionally	137 (67.5)	40 (22.0)	177 (46.0)
	Never	46 (22.7)	69 (37.9)	115 (29.8)
Frequency of hand washing	Every hour	10 (4.9)	11 (6.0)	21 (5.5)
	After every cleaning task	189 (93.1)	171 (94.0)	360 (93.5)
	Only after used restroom and before eating	2 (1.0)	0	2 (0.5)
	Only when hands are visibly dirty	2 (1.0)	0	2 (0.5)
Hand cleaning soon after handling waste materials	Yes	163 (80.3)	170 (93.4)	333 (86.5)
	Sometimes	37 (18.2)	12 (6.6)	49 (12.7)
	No	3 (1.5)	0	3(0.8)
Method of hand washing	Only with water	57 (28.1)	17 (9.3)	74 (19.2)
	With soap and water	146 (71.9)	163 (89.6)	309 (80.3)
	With alcohol based sanitizer	0	2 (1.1)	2 (0.5)
Fingernail status	Trimmed	137 (67.5)	149 (81.9)	286 (74.3)
	Non-trimmed	66 (32.5)	33 (18.1)	99 (25.7)
Safe toilet facility at home	Yes	174 (85.7)	167 (91.8)	341 (88.6)
	No	29 (14.3)	15 (8.2)	44 (11.4)
Hand hygiene practice at home	Yes	177 (87.2)	178 (97.8)	355 (92.2)
	Sometimes	19 (9.4)	4 (2.2)	23 (6.0)
	No	7 (3.4)	0	7 (1.8)
Source of water for drinking	Tap water	185 (91.1)	165 (90.7)	350 (90.9)
	Hand dug well water	18 (8.9)	17 (9.3)	35 (9.1)

**Abbreviations**: PPE = Personnel protective equipment; IPC = Infection prevention and control.

### Behavioral and clinical characteristics of study participants

A higher proportion of hospital janitors reported use of prescribed antibiotics in the last three months, 100 (49.3%) compared to 50 (27.5%), non-hospital janitors. Furthermore, a greater percentage of hospital janitors had a history of gastrointestinal (GI) symptom in the last three months, 74 (36.5%) compared to 26 (14.3%), non-hospital janitors. A similar disparity was observed for a history of urinary tract infection (UTI) in the last three months, with hospital janitors 59 (29.1%), reporting a higher proportion than 23 (12.6%), non-hospital janitors (Table 3).

**Table 3:** Behavioral and clinical related characteristics of hospital (N = 203) and non-hospital janitors (N = 182) at the UoG, Northwest Ethiopia, June to September 2025.

Variables	Categories		Study Participants		Total (N = 385); n (%)
			Hospital Janitors (N = 203); n (%)	Non-hospital Janitors (N = 182); n (%)	
Antibiotics used without prescription in the last 3 months	Yes	< 1 week	34 (59.6)	37 (66.1)	71 (62.8)
		1-2 weeks	18 (31.6)	19 (33.9)	37 (32.8)
		> 2 weeks	5 (8.8)	0	5 (4.4)
		Total	57 (28.1)	56 (30.8)	113 (29.4)
	No		146 (71.9)	126 (69.2)	272 (70.6)
Drinking unpasteurized milk	Yes		60 (29.6)	44 (24.2)	104 (27.0)
	No		143 (70.4)	138 (75.8)	281 (72.9)
Eating raw meat	Yes		36 (17.7)	27 (14.8)	63 (16.4)
	No		167 (82.3)	155 (85.2)	322 (83.6)
Eating uncooked vegetables	Yes		76 (37.4)	43 (23.6)	119 (30.9)
	No		127 (62.6)	139 (76.4)	266 (69.1)
Used prescribed antibiotics in the last 3 months	Yes	Appropriately	30 (30.0)	27 (54.0)	57 (38.0)
		Not- appropriately	70 (70.0)	23 (46.0)	93 (62.0)
		Total	100 (49.3)	50 (27.5)	150 (39.0)
	No		103 (50.7)	132 (72.5)	235 (61.0)
History of GI symptom in the last 3 months	Yes		74 (36.5)	26 (14.3)	100 (26.0)
	No		129 (63.5)	156 (85.7)	285 (74.0)
History of UTI in the last 3 months	Yes		59 (29.1)	23 (12.6)	82 (21.3)
	No		144 (70.9)	159 (87.4)	303 (78.7)
History of chronic disease	Yes		14 (6.9)	17 (9.3)	31(8.1)
	No		189 (93.1)	165 (90.7)	354 (91.9)
History of hospital admission in the last 3 months	Yes	Medical instrumentation	4 (11.4)	15 (71.4)	19 (33.9)
		No- Medical instrumentation	31 (88.6)	6 (28.6)	37 (66.1)
		Total	35 (17.2)	21 (11.5)	56 (14.5)
	No		168 (82.8)	161 (88.5)	329 (85.5)
Close contact with hospitalized person in the last 3 months	Yes		101 (49.7)	60 (33.0)	161 (41.8)
	No		102 (50.3)	122 (67.0)	224 (58.2)

**Abbreviations:** GI = Gastrointestinal; UTI = Urinary tract infection.

### Fecal carriage rate of ESBL and Carbapenemase-producing Enterobacteriaceae among hospital and non-hospital janitors

Among 385 janitors, a total of 416 isolates were recovered (229 from hospital janitors and 187 from non-hospital janitors). Single-organism colonization occurred in 354 janitors, while 31 janitors (26 hospital, 5 non-hospital) had co-colonization with two organisms. *Escherichia coli* was the predominant organism in both groups, constituting 124 (54.2%) of isolates from hospital janitors and 125 (66.8%) from non-hospital janitors.

The overall fecal carriage rate of ESBL-PE among janitors was 21.6% (95% CI: 17.6, 26.0). This rate was significantly higher in hospital janitors compared to non-hospital janitors ((28.6% (95% CI: 22.5, 35.3) vs. 13.7% (95% CI: 9.1, 19.6), respectively; Chi-square = 12.5, *p*-value < 0.001)). Similarly, the overall fecal carriage rate of CPE was 3.6% (95% CI: 2.0, 6.0), with hospital janitors again showing a significantly higher prevalence than non-hospital janitors ((5.9% (95% CI: 3.1, 10.1) vs. 1.1% (95% CI: 0.1, 3.9), respectively; Chi-square = 6.3, *p*-value = 0.012)).

Among the total bacterial isolates from hospital and non-hospital janitors, the prevalence of ESBL-PE was higher in hospital janitors compared to non-hospital janitors (25.3% vs. 13.4%, respectively). Similarly, phenotypic detected carbapenemase-producing isolates were also more prevalent among hospital janitors than non-hospital janitors (5.2% vs. 1.1%, respectively). Additionally, 2.6% and 0.5% of isolates from hospital and non-hospital janitors, respectively, were both ESBL- and carbapenemase-producing. Furthermore, the co-occurrence of resistance phenotypes was more common in the hospital group. Among hospital janitors, 94.8% of ESBL-PE isolates were classified as MDR, compared to 80.0% of ESBL-PE isolates from non-hospital janitors. All CPE isolates from both groups were MDR (Table 4).

**Table 4:** Fecal carriage rate of ESBL and Carbapenemase-producing Enterobacteriaceae among hospital (N = 203) and non-hospital janitors (N = 182) at the UoG, Northwest Ethiopia, June to September 2025.

Enterobacteriaceae isolates (N = 416)	Study participants	Proportion from total Enterobacteriaceae isolates (N = 416)					Proportion from total study participants(HJ = 203; NHJ = 182)	
		ESBL-producing (N (%))	Carbapenemase-producing (N (%))	Both ESBL and Carbapenemase-producing (N (%))	Both ESBL and MDR positive (N (%))	Both Carbapenemase and MDR positive (N (%))		
							ESBL-producing (N (%))	Carbapenemase-producing (N (%))
*E. coli* (N = 249)	HJ = 124	38 (30.6)	2 (1.6)	1 (0.8)	35 (28.2)	2 (1.6)	38 (18.7)	2 (1.0)
	NHJ = 125	22 (17.6)	0	0	17 (13.6)	0)	22 (12.1)	0
*K. pneumoniae* (N = 35)	HJ = 22	7 (31.8)	7 (31.8)	3 (13.6)	7 (31.8)	7 (31.8)	7 (3.4)	7 (3.4)
	NH = 13	2 (15.4)	0	0	2 (15.4)	0	2 (1.1)	0
*K. oxytoca* (N = 20)	HJ = 13	5 (38.5)	1 (7.7)	1 (7.7)	5 (38.5)	1 (7.7)	5 (2.5)	1 (0.5)
	NHJ = 7	0	0	0	0	0	0	0
*K. ozaenae* (N = 14)	HJ = 11	4 (36.4)	1 (9.1)	1 (9.1)	4 (36.4)	1 (9.1)	4 (2.0)	1 (0.5)
	NHJ = 3	0	1 (33.3)	0	0	1 (33.3)	0	1 (0.6)
*E. cloacae* (N = 11)	HJ = 10	2 (20.0)	1 (10.0)	0	2 (20.0)	1 (10.0)	2 (1.0)	1 (0.5)
	NHJ = 1	0	0	0	0	0	0	0
*E. aerogens* (N = 7)	HJ = 6	1 (16.7)	0	0	1 (16.7)	0	1 (0.5)	0
	NHJ = 1	0	0	0	0	0	0	0
*C. koseri* (N = 34)	HJ = 13	0	0	0	0	0	0	0
	NHJ = 21	0	0	0	0	0	0	0
*C. freundii* (N = 7)	HJ = 4	0	0	0	0	0	0	0
	NHJ = 3	0	0	0	0	0	0	0
*Shigella* species (N = 9)	HJ = 6	0	0	0	0	0	0	0
	NHJ = 3	0	0	0	0	0	0	0
*Proteus* species (N = 8)	HJ = 8	0	0	0	0	0	0	0
*P. rettgeri* (N = 8)	HJ = 2	0	0	0	0	0	0	0
	NHJ = 6	1 (16.7)	1 (16.7)	1 (16.7)	1 (16.7)	1 (16.7)	1 (0.6)	1 (0.6)
*P. stuartii* (N = 12)	HJ = 9	1 (11.1)	0	0	1 (11.1)	0	1 (0.5)	0
	NHJ = 3	0	0	0	0	0	0	0
*Serratia* species (N = 2)	HJ = 1	0	0	0	0	0	0	0
	NHJ = 1	0	0	0	0	0	0	0
Total (N = 416)	HJ = 229	**58 (25.3)**	**12 (5.2)**	**6 (2.6)**	**55 (24.0)**	**12 (5.2)**	**58 (28.6)**	**12 (5.9)**
	NHJ = 187	**25 (13.4)**	**2 (1.1)**	**1 (0.5)**	**20 (10.7)**	**2 (1.1)**	**25 (13.7)**	**2 (1.1)**

**Key:** HJ=Hospital Janitors; NHJ=Non-hospital Janitors.

**Abbreviations:** MDR = Multidrug-resistant; ESBL-PE = Extended-spectrum Beta-lactamase-producing Enterobacteriaceae; CPE = Carbapenemase- producing Enterobacteriaceae.

### Proportion of ESBL and carbapenemase-producing Enterobacteriaceae isolates among hospital and non-hospital janitors

**Fig 2:**
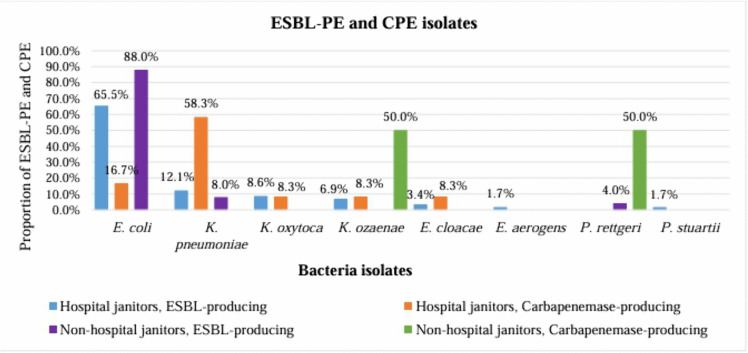
Proportion of ESBL and Carbapenemase-producing Enterobacteriaceae isolates among hospital (N = 203) and non-hospital janitors (N = 182) at the UoG, Northwest Ethiopia, June to September 2025.

Analysis of the predominant bacterial species revealed that *E. coli* was the most common ESBL-producing isolate in both janitor groups, accounting for 65.5% (38/58) and 88.0% (22/25) of isolates from hospital and non-hospital janitors, respectively. However, among hospital janitors, the leading carbapenemase-producing isolate was *K. pneumoniae*, at 58.3% (7/12) (Fig 2).

### Antimicrobial resistance patterns of ESBL-producing Enterobacteriaceae isolates from hospital and non-hospital janitors

The AMR pattern of ESBL-producing isolates demonstrated high resistance in both groups, with isolates from hospital janitors generally exhibiting higher rates. Resistance to ceftazidime, ceftriaxone, and cefotaxime was 100% in isolates from hospital janitors and 92.0% in isolates from non-hospital janitors. Among isolates from hospital janitors, resistance was 91.4% to tetracycline, 82.8% to gentamicin, and 74.1% to sulfamethoxazole-trimethoprim. In comparison, isolates from non-hospital janitors showed lower resistance rates to tetracycline (80.0%), gentamicin (60.0%), and sulfamethoxazole-trimethoprim (56.0%).

Notably, resistance to several critical antibiotics was more pronounced among isolates from the hospital janitors. Among hospital janitors, 22.4% of isolates were resistant to meropenem, 19.0% to ciprofloxacin, and 12.1% to amoxicillin-clavulanic acid. In contrast, isolates from non-hospital janitors showed lower resistance to these agents, with rates of 8.0% for meropenem, 16.0% for ciprofloxacin, and 16.0% for amoxicillin-clavulanic acid. Additionally, 16.0% of non-hospital janitor isolates were resistant to imipenem, while only 4.0% were resistant to amikacin (Table 5).

**Table 5:** Antimicrobial resistance patterns of ESBL-producing Enterobacteriaceae isolates from hospital (N = 203) and non-hospital janitors (N = 182) at the UoG, Northwest Ethiopia, June to September 2025.

Antibiotics	Patterns	ESBL-PE isolates from hospital janitors (N = 58)								ESBL-PE isolates from non-hospital janitors (N = 25)			
		*E. coli* (N = 38); N (%)	*K. pneumoniae* (N = 7); N (%)	*K. oxytoca* (N = 5); N (%)	*K. ozaenae* (N = 4); N (%)	*E. cloacae* (N = 2); N (%)	*E. aerogens* (N = 1); N (%)	*P. stuartii* (N=); N (%)	Total N (%)	*E. coli* (N = 22); N (%)	*K. pneumoniae* (N = 2); N (%)	*P. rettgeri* (N = 1); N (%)	Total N (%)
CAZ	S	0	0	0	0	0	0	0	0	2(9.1)	0	0	2(8.0)
	R	38(100.0)	7(100.0)	5(100.0)	4(100.0)	2(100.0)	1(100.0)	1(100.0)	**58(100.0)**	20(90.9)	2(100.0)	1(100.0)	**23(92.0)**
CRO	S	0	0	0	0	0	0	0	0	2(9.1)	0	0	2(8.0)
	R	38(100.0)	7(100.0)	5(100.0)	4(100.0)	2(100.0)	1(100.0)	1(100.0)	**58(100.0)**	20(90.9)	2(100.0)	1(100.0)	**23(92.0)**
CTX	S	0	0	0	0	0	0	0	0	2(9.1)	0	0	2(8.0)
	R	38(100.0)	7(100.0)	5(100.0)	4(100.0)	2(100.0)	1(100.0)	1(100.0)	**58(100.0)**	20(90.9)	2(100.0)	1(100.0)	**23(92.0)**
CXT	S	20(52.6)	3(42.9)	4(80.0)	4(100.0)	1(50.0)	1(100.0)	1(100.0)	34(58.6)	17(77.3)	2(100.0)	0	19(76.0)
	R	18(47.4)	4(57.1)	1(20.0)	0	1(50.0)	0	0	**24(41.4)**	5(22.7)	0	1(100.0)	**6(24.0)**
AMC	S	32(84.2)	7(100.0)	5(100.0)	3(75.0)	2(100.0)	1(100.0)	1(100.0)	51(87.9)	21(95.5)	0	0	21(84.0)
	R	6(15.8)	0	0	1(25.0)	0	0	0	**7(12.1)**	1(4.5)	2(100.0)	1(100.0)	**4(16.0)**
MER	S	32(84.2)	2(28.6)	4(80.0)	3(75.0)	2(100.0)	1(100.0)	1(100.0)	45(77.6)	22(100.0)	1(50.0)	0	23(92.0)
	R	6(15.8)	5(71.4)	1(20.0)	1(25.0)	0	0	0	**13(22.4)**	0	1(50.0)	1(100.0)	**2(8.0)**
IMP	S	26(68.4	2(28.6)	4(80.0)	2(50.0)	1(50.0)	0	1(100.0)	36(62.1)	20(90.9)	1(50.0)	0	21(84.0)
	R	12(31.6)	5(71.4)	1(20.0)	2(50.0)	1(50.0)	1(100.0)	0	**22(37.9)**	2(9.1)	1(50.0)	1(100.0)	**4(16.0)**
GEN	S	8(21.1)	2(28.6)	0	0	0	0	0	10(17.2)	10(45.5)	0	0	10(40.0)
	R	30(78.9)	5(71.4)	5(100.0)	4(100.0)	2(100.0)	1(100.0)	1(100.0)	**48(82.8)**	12(54.5)	2(100.0)	1(100.0)	**15(60.0)**
AK	S	38(100.0)	0	4(80.0)	0	0	1(100.0)	0	43(74.1)	22(100.0)	2(100.0)	0	24(96.0)
	R	0	7(100.0)	1(20.0)	4(100.0)	2(100.0)	0	1(100.0)	**15(25.9)**	0	0	1(100.0)	**1(4.0)**
TET	S	5(13.2)	0	0	0	0	0	0	5(8.6)	5(22.7)	0	0	5(20.0)
	R	33(86.8)	7(100.0)	5(100.0)	4(100.0)	2(100.0)	1(100.0)	1(100.0)	**53(91.4)**	17(77.3)	2(100.0)	1(100.0)	**20(80.0)**
C	S	23(60.5)	4(57.1)	2(40.0)	2(50.0)	1(50.0)	1(100.0)	1(100.0)	34(58.6)	16(72.7)	1(50.0)	0	17(68.0)
	R	15(39.5)	3(42.9)	3(60.0)	2(50.0)	1(50.0)	0	0	**24(41.4)**	6(27.3)	1(50.0)	1(100.0)	**8(32.0)**
CIP	S	31(81.6)	4(57.1)	4(80.0)	4(100.0)	2(100.0)	1(100.0)	1(100.0)	47(81.0)	20(90.9	1(50.0)	0	21(84.0)
	R	7(18.4)	3(42.9)	1(20.0)	0	0	0	0	**11(19.0)**	2(9.1)	1(50.0)	1(100.0)	**4(16.0)**
SXT	S	12(31.6)	2(28.6)	0	0	0	1(100.0)	0	15(25.9)	10(45.5)	1(50.0)	0	11(44.0)
	R	26(68.4)	5(71.4)	5(100.0)	4(100.0)	2(100.0)	0	1(100.0)	**43(74.1)**	12(54.5)	1(50.0)	1(100.0)	**14(56.0)**

**Key**: S=Sensitive; R=Resistance.

**Abbreviations**: ESBL-PE = Extended-spectrum Beta-lactamase-producing Enterobacteriaceae; CAZ = Ceftazidime; CRO = Ceftriaxone; CTX = Cefotaxime; CXT = Cefoxitin; AMC = Amoxicillin-clavulanic acid; MER = Meropenem; IMP = Imipenem; GEN = Gentamycin; AK = Amikacin; TET = Tetracycline; CHL = Chloramphenicol; CIP = Ciprofloxacin; SXT = Sulphamethoxazole-trimethoprim.

### Antimicrobial resistance patterns of carbapenemase-producing Enterobacteriaceae isolates from hospital and non-hospital janitors

All CPE isolates from both janitor groups were resistant to meropenem, gentamicin, and tetracycline. Furthermore, all CPE isolates from non-hospital janitors were also resistant to ceftazidime, ceftriaxone, cefotaxime, imipenem, and chloramphenicol (**Table 6**).

**Table 6:** Antimicrobial resistance patterns of carbapenemase-producing Enterobacteriaceae isolates from hospital (N = 203) and non-hospital janitors (N = 182) at the UoG, Northwest Ethiopia, June to September 2025.

Antibiotics	Patterns	CPE isolates from hospital janitors (N = 12)						CPE isolates from non-hospital janitors (N = 2)		
		*E. coli* (N = 2)	*K. pneumoniae* (N = 7)	*K. oxytoca* (N = 1)	*K. ozaenae* (N = 1)	*E. cloacae* (N = 1)	Total N (%)	*K. ozanae* (N = 1)	*P. rettgeri* (N = 1)	Total N (%)
CAZ	S	0	2	0	0	1	3(25.0)	0	0	0
	R	2	5	1	1	0	**9(75.0)**	1	1	**2(100.0)**
CRP	S	0	1	0	0	1	2(16.7)	0	0	0
	R	2	6	1	1	0	**10(83.3)**	1	1	**2(100.0)**
CTX	S	1	2	0	0	1	4(33.3)	0	0	0
	R	1	5	1	1	0	**8(66.7)**	1	1	**2(100.0)**
CXT	S	0	3	1	1	1	6(50.0)	1	0	1(50.0)
	R	2	4	0	0	0	**6(50.0)**	0	1	**1(50.0)**
AMC	S	1	5	1	0	1	8(66.7)	1	0	1(50.0)
	R	1	2	0	1	0	**4(33.3)**	0	1	**1(50.0)**
MER	S	0	0	0	0	0	0	0	0	0
	R	2	7	1	1	1	**12(100.0)**	1	1	**2(100.0)**
IMP	S	1	1	0	0	0	2(16.7)	0	0	0
	R	1	6	1	1	1	**10(83.3)**	1	1	**2(100.0)**
GEN	S	0	0	0	0	0	0	0	0	0
	R	2	7	1	1	1	**12(100.0)**	1	1	**2(100.0)**
AK	S	1	1	0	0	1	3(25.0)	1	0	1(50.0)
	R	1	6	1	1	0	**9(75.0)**	0	1	**1(50.0)**
TET	S	0	0	0	0	0	0	0	0	0
	R	2	7	1	1	1	**12(100.0)**	1	1	**2(100.0)**
CHL	S	1	3	0	0	1	5(41.7)	0	0	0
	R	1	4	1	1	0	**7(58.3)**	1	1	**2(100.0)**
CIP	S	1	4	1	1	1	8(66.7)	1	0	1(50.0)
	R	1	3	0	0	0	**4(33.3)**	0	1	**1(50.0)**
SXT	S	0	2	0	0	1	3(25.0)	1	0	1(50.0)
	R	2	5	1	1	0	**9(75.0)**	0	1	**1(50.0)**

**Key**: S=Sensitive; R=Resistance.

Abbreviations: CPE = carbapenemase-producing Enterobacteriaceae; CAZ = Ceftazidime; CRO = Ceftriaxone; CTX = Cefotaxime; CXT = Cefoxitin; AMC = Amoxicillin-clavulanic acid; MER = Meropenem; IMP = Imipenem; GEN = Gentamycin; AK = Amikacin; TET = Tetracycline; CHL = Chloramphenicol; CIP = Ciprofloxacin; SXT = Sulphamethoxazole-trimethoprim.

### Distribution of ESBL and Carbapenemase-producing Enterobacteriaceae isolates among eight working sites of hospital janitors at the UoGCSH

**Fig 3:**
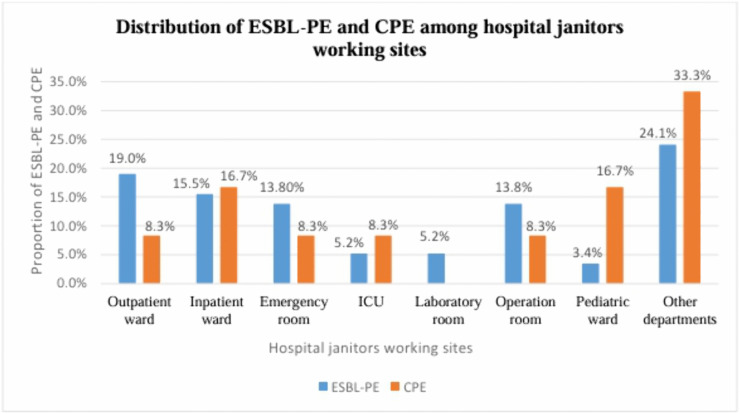
Distribution of ESBL-PE and CPE isolates among hospital janitors at the UoGCSH working sites, Northwest Ethiopia, June to September 2025 (N = 203).

The distribution of resistant isolates across hospital working sites is detailed in (Fig 3). Among the 58 ESBL-producing isolates, the highest proportion (24.1%) was identified in other departments, followed by the outpatient ward (19.0%), inpatient ward (15.5%), emergency room (13.8%), and operation room (13.8%). A similar distribution was observed for the 12 carbapenemase-producing isolates, with the highest prevalence also found in other departments (33.3%), followed by the inpatient and pediatric wards (16.7% each).

### Distribution of ESBL-PE and CPE among non-hospital janitors across three UoG Campuses

**Fig 4:**
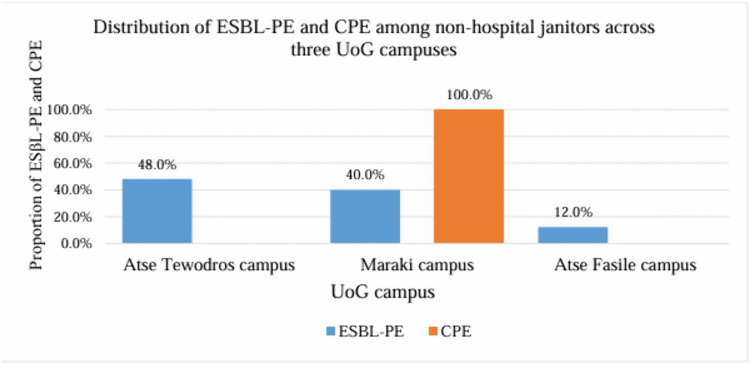
Distribution of ESBL-PE and CPE isolates among non-hospital janitors across three UoG campuses, Northwest Ethiopia, June to September 2025 (N = 182).

The distribution of resistant isolates across the UoG campuses is detailed in (Fig 4). Among the 25 ESBL-producing isolates, the highest proportion (48.0%) was identified at Atse Tewodros campus, followed by the Maraki campus (40.0%). Both of the two CPE isolates were identified at Maraki campus.

### Factors associated with fecal carriage of ESBL-PE among study participants

Variables with a *p*-value of ≤0.25 in the bivariable analysis were included in the multivariable logistic regression model. The final model identified three factors significantly associated with ESBL-PE carriage among hospital janitors and two factors among non-hospital janitors. Among hospital janitors, a lack of hand hygiene practice at home (AOR: 16.39; 95% CI: 2.14, 125.50), antibiotic use within the past three months (AOR: 3.54; 95% CI: 1.52, 8.23), and a history of UTI in the past three months (AOR: 4.93; 95% CI: 2.17, 11.20) were also significantly associated with increased odds of ESBL-PE carriage (Table 7).

**Table 7:** Factors associated with fecal carriage of ESBL-PE among hospital janitors at the UoG, Northwest Ethiopia, June to September 2025 (N = 203).

Variables	Category	ESBL-PE N (%)		Bivariable analysis		Multivariable analysis	
		Positive (N = 58)	Negative (N = 145)	COR (95% CI)	*P*-value	AOR (95% CI)	*P*- value
Age	18-28	15 (20.0%)	60 (80.0%)	1		1	
	29-39	41 (36.3%)	72 (63.7%)	2.28 (1.15, 4.51)	0.018	1.35 (0.53, 3.45)	0.533
	≥40	2 (13.3%)	13 (86.7%)	0.62 (0.13, 3.03)	0.550	0.21 (0.03, 1.58)	0.129
Service years	1-5	17 (21.5%)	62 (78.5%)	1		1	
	>5	41 (33.1%)	83 (66.9%)	1.80 (0.94, 3.47)	0.078	0.93 (0.36, 2.39)	0.878
Exposure to contaminated surfaces	Frequently	4 (20.0%)	16 (80.0%)	0.43 (0.12, 1.49)	0.181	0.23 (0.05, 1.06)	0.059
	Occasionally	37 (27.0)%	100 (73.0%)	0.63 (0.31, 1.28)	0.202	0.56 (0.23, 1.38)	0.211
	Never	17 (37.0%)	29 (63.0%)	1		1	
Hand hygiene practice at home	Yes	47 (26.6%)	130 (73.4%)	1		1	
	Sometimes	6 (31.6%)	13 (68.4%)	1.28 (0.46, 3.55)	0.640	1.31 (0.35, 4,95)	0.691
	No	5 (71.4%)	2 (28.6%)	6.92 (1.30, 36.86)	0.024	**16.39 (2.14, 125.50**	0.007*
Antibiotics used without prescription in the last 3 month	Yes	21 (36.8%)	36 (63.2%)	1.72 (0.89, 3.31)	0.105	0.95 (0.40, 2.26)	0.915
	No	37 (25.3%)	109 (74.7%)	1		1	
Drinking unpasteurized milk	Yes	21 (35.0%)	39 (65.0%)	1.54 (0.81, 2.95)	0.191	1.01 (0.44, 2.28)	0.990
	No	37 (25.9%)	106 (74.1%)	1		1	
Prescribed antibiotics used in the last 3 month	Yes	44 (44.0%)	56 (56.0%)	5.00 (2.51, 9.94)	<0.001	**3.54 (1.52, 8.23)**	0.003*
	No	14 (13.6%)	89 (86.4%)	1		1	
History of GI symptom in the last 3 month	Yes	30 (40.5%)	44 (59.5%)	2.46 (1.32, 4.60)	0.005	1.78 (0.81, 3.93)	0.154
	No	28 (21.7%)	101 (78.3%)	1		1	
History of UTI symptom in the last 3 month	Yes	31 (52.5%)	28 (47.5%)	4.80 (2.48, 9.29)	<0.001	**4.93 (2.17, 11.20)**	0.001*
	No	27 (18.7%)	117 (81.3%)	1		1	
History of hospital admission in the last 3 month	Yes	13 (37.1%)	22 (62.9%)	1.62 (0.75, 3.47)	0.220	1.52 (0.51, 4.58)	0.453
	No	45 (26.8%)	123 (73.2%)	1		1	
Close contact with hospitalized person in the last 3 month	Yes	34 (33.7%)	67 (66.3%)	1.65 (0.89, 3.05)	0.111	1.40 (0.64, 3.05)	0.403
	No	24 (23.5%)	78 (76.5%)	1		1	

**Keys**: **Bold** and * = Statistically significant at *p*-value <0.05; 1 = Reference group.

**Abbreviations**: ESBL-PE = Extended-spectrum Beta-lactamase-producing Enterobacteriaceae; COR = Crude odds ratio; AOR = Adjusted odds ratio; CI = Confidence interval; UTI = Urinary tract infection; GI = Gastrointestinal.

Among non-hospital janitors, Age ≥ 40 years was a significant predictor of ESBL-PE carriage (AOR: 6.38; 95% CI: 1.03, 39.40). Furthermore, antibiotic use without a prescription within the past three months was also significantly associated with increased odds of ESBL-PE carriage (AOR: 4.79; 95% CI: 1.72, 13.33). A lack of hand hygiene practice at home and age ≥ 40 years, should be interpreted with caution due to the wide CIs (Table 8).

**Table 8:** Factors associated with fecal carriage of ESBL-PE among non-hospital janitors at the UoG, Northwest Ethiopia, June to September 2025 (N = 182).

Variables	Category	ESBL-PE N (%)		Bivariable analysis		Multivariable analysis	
		Positive (N = 25)	Negative (N = 157)	COR (95% CI)	P-value	AOR (95% CI)	P-value
Age	18-28	14 (12.0%)	103 (88.0%)	1		1	
	29-39	7 (12.5%)	49 (87.5%)	1.05 (0.40, 2.77)	0.920	0.94 (0.32, 2.82)	0.916
	≥40	4 (44.4%)	5 (55.6%)	5.87 (1.41, 24.56)	0.015	**6.38 (1.03, 39.40)**	0.046*
UoG campuses	Atse Tewodros campus	12 (13%)	80 (87.0%)	1.65 (0.44, 6.23)	0.460	2.03 (0.38, 10.96)	0.410
	Maraki campus	10 (18.5%)	44 (81.5%)	2.50 (0.64, 9.81)	0.189	1.73 (0.28, 10.60)	0.554
	Atse Fasile campus	3 (8.3%)	33 (91.7%)	1		1	
Antibiotics used without prescription in the last 3 months	Yes	17 (30.4%)	39 (69.6%)	6.43 (2.58, 16.05)	<0.001	**4.79 (1.72, 13.33)**	0.003*
	No	8 (6.4%)	118 (93.6%)	1		1	
Exposure to contaminated surfaces	Frequently	11 (15.1%)	62 (84.9%)	2.27 (0.75, 6.91)	0.149	1.05 (0.30, 3.69)	0.945
	Occasionally	9 (22.5%)	31 (77.5%)	3.72 (1.15, 12.03)	0.028	3.51 (0.78, 15.78)	0.102
	Never	5 (7.3%)	64 (92.7%)	1		1	
Prescribed antibiotics used in the last 3 month	Yes	14 (28.0%)	36 (72.0%)	4.28 (1.79, 10.24)	0.001	2.74 (0.98, 7.63)	0.054
	No	11 (8.3%)	121 (91.7%)	1		1	
Close contact with hospitalized person in the last 3 months	Yes	13 (21.7%)	47 (78.3%)	2.54 (1.08, 5.97)	0.033	1.82 (0.61, 5.44)	0.286
	No	12 (9.8%)	110 (90.2%)	1		1	

**Keys**: **Bold** and * = Statistically significant at *P*-value <0.05; 1 = Reference group.

**Abbreviations**: ESBL-PE = Extended-spectrum Beta-lactamase-producing Enterobacteriaceae; UoG = University of Gondar; COR = Crude odds ratio; AOR = Adjusted odds ratio; CI = Confidence interval.

## Discussion

Fecal carriage of ESBL and carbapenemase-producing Enterobacteriaceae is a growing public health threat worldwide, contributing to both subsequent infections in carriers and potential person-to-person transmission [47,48]. Over the past decade, the prevalence of ESBL-PE and CPE has risen dramatically across the globe, including Ethiopia [18–20,49–51]. Despite this trend, data on fecal carriage rates among janitors in Ethiopia remain scarce.

In the present study, the fecal carriage rate of ESBL-PE among hospital janitors was 28.6% (95% CI: 22.5, 35.3), aligning with findings from food handlers in Gondar (21.73%) and Dilla (25.3%), Ethiopia [19,20]. However, it is notably lower than rates reported among HCWs in Vietnam (65%) [52], Madagascar (45.0%) [53], and caregivers in Rwanda (37%) [54]. This variation in carriage rates likely reflects distinct microbial ecologies and selective pressures, including local antibiotic use patterns influencing resistance gene pools, variations in strain virulence and colonization fitness, and differing levels of occupational exposure to antimicrobial residues and resistant bacteria in the environment [55,56]. Conversely, the rate in our study was higher than those reported among janitors in rehabilitation units in Israel, Italy, France, and Spain (5.5%) [57], as well as among HCWs in Egypt (18.5%) [58], Spain (3.1%) [59], and Switzerland (5.4%) [60]. This elevated carriage may be due to factors such as irrational antibiotic use, environmental hygiene, inadequate hospital sanitation, and high prevalence of ESBL-PE in the study setting [61–63]**.** This may also be explained by occupational exposure to contaminated surfaces, biomedical waste, and patient-care environments, combined with frequent hand contact with high-touch areas [45]. This finding indicating that hospitals should integrate janitors into antimicrobial stewardship and IPC programs and provide regular training on hand hygiene and personnel protective equipment (PPE) use.

The ESBL-PE carriage rate among non-hospital janitors in this study was 13.7% (95% CI: 9.1, 19.6). This finding is consistent with community studies in Madagascar (10.1%) [64], and among healthcare students in Portugal (14.4%) [65]. However, it is lower than rates reported for food handlers in Gondar (21.73%) and Dilla (25.3%), Ethiopia [19,20], and community studies in Addis Ababa, Ethiopia (31.4%) [18], and Burkina Faso (22.0%) [66].Conversely, our rate is higher than those found among food handlers in Gambia (5.0%) [67], and Qatar (1.5%) [68], and community population in Morocco (4.3%) [69]. These variations highlight how carriage rates are governed by regional differences in the dissemination of specific resistance plasmids and bacterial clones, the intensity of exposure to contaminated reservoirs, and the technical sensitivity of the culture-based detection protocols employed [48,70]. This study demonstrates that, *E. coli* was a leading ESBL-PE, accounting for 65.5% and 88.0% of ESBL-PE isolates from hospital and non-hospital janitors, respectively. This predominance agrees with study findings from food handlers in Gondar and Dilla, Ethiopia [19,20], and community studies in Addis Ababa, Ethiopia [18], and Burkina Faso [66].This bacteria is highly efficient at acquiring and transferring resistance genes via mobile genetic elements like plasmids within the GI tract. This allows resistance traits to disseminate not only among *E. coli* strains but also to other bacterial species, creating a reservoir of AMR organisms in carriers [71].

The fecal carriage rate of CPE among hospital janitors participated in this study was 5.9% (95% CI: 3.1, 10.1). This finding is consistent with studies among parturient women in Gabon (4.6%) [72], food handlers in Kuwait (7.7%) [73], and community populations in India (6.1%) [49]. However, it is higher than rates reported among food handlers in Gondar, Ethiopia (2.4%) [20], community studies in Addis Ababa, Ethiopia (0.8%) [18], and China (0.5%) [74], and HCWs in Switzerland (0.1%) [60]. The variation in the prevalence rates could be due to Carbapenem selection pressure within healthcare environments, heterogeneous occupational exposure to hospital reservoirs and colonized patients [75,76]. The findings of this study indicate that, *K. pneumoniae* was the leading CPE isolated from hospital janitors, accounting for 58.3% of CPE isolates. This aligns with findings from food handlers in Gondar, Ethiopia [20], and community study in Addis Ababa, Ethiopia [18]. In contrast, *E. coli* has been reported as the common carbapenemase producer among parturient women in Gabon [72], and community study in India [49]. This regional variation in the dominant CPE species likely reflects differences in local antibiotic prescribing practices [77], hospital infection control protocols [78], and the specific prevalence and transmissibility of resistance plasmids within distinct bacterial populations [70,79].

The CPE carriage rate among non-hospital janitors participated in the present study was 1.1% (95% CI: 0.1, 3.9). This finding aligns with studies on food handlers in Gondar, Ethiopia (2.4%) [20], community populations in Addis Ababa, Ethiopia (0.8%) [18], and China (0.5%) [74], and HCWs in Switzerland (0.1%) [60]. However it was lower than studies conducted among parturient women in Gabon (4.6%) [72], food handlers in Kuwait (7.7%) [73], and community populations in India (6.1%) [49]. This disparity highlights the influence of the variable community-level antibiotic selection pressure, differences in exposure to potential environmental or human reservoirs, and the geographical circulation of distinct resistant lineages [70,80].

This study demonstrates that, amoxicillin-clavulanic acid (87.9%) and amikacin (96.0%) demonstrated better in *vitro* performance against ESBL-PE isolates from hospital and non-hospital janitors, respectively. This high susceptibility is likely attributable to the mechanisms of action of these drugs. Amikacin, an aminoglycoside, retains effectiveness against many ESBL-PE because its activity is not inhibited by ESBL enzymes [81]. Similarly, the clavulanic acid, component of amoxicillin-clavulanic acid can inhibit many ESBL enzymes, restoring the activity of the amoxicillin component in many isolates [82]. However, our result regarding amoxicillin-clavulanic acid differs from studies among food handlers in Gondar, Ethiopia [20], and community populations in Addis Ababa, Ethiopia [18], which reported lower susceptibility. According to CLSI guidelines, all confirmed ESBL-PE must be reported as resistant to all penicillins (including amoxicillin-clavulanic acid), regardless of *in vitro* susceptibility results, due to documented therapeutic failure and poor clinical outcomes. Our previously reported *in vitro* percentages (87.9% for amoxicillin-clavulanic acid) are retained only as epidemiological surveillance data and should not be used to guide clinical therapy [40]. Regarding MDR, our study reported that 94.8% of ESBL-PE isolates from hospital janitors and 80.0% from non-hospital janitors were identified as MDR. This higher MDR among ESBL-PE is primarily due to the location of resistance genes on plasmids. Plasmids often carry multiple resistance genes for different antibiotic classes, and they can efficiently transfer this entire MDR profile to other bacteria through conjugation [83].

This study found that the observed fecal carriage rates of ESBL-PE (28.6% vs. 13.7%), and CPE (5.9% vs. 1.1%), among hospital janitors compared to non-hospital janitors, showed significant difference in bivariable analysis of Chi-square test (*p* < 0.001 and *p* = 0.012, respectively). The significantly higher fecal carriage rates of ESBL-PE and CPE among hospital janitors, suggest a potential association with occupational setting. Hospitals are reservoirs for antibiotic-resistant bacteria, and janitors are routinely exposed to contaminated surfaces, medical waste, and high-touch areas in clinical settings while performing cleaning duties. This constant occupational exposure increases their risk of acquiring and being colonized by MDR-organisms compared to janitors working in non-hospital settings with lower pathogen density and antibiotic selective pressure. While our cross-sectional design precludes causal attribution and we lack environmental or molecular evidence to trace specific isolates to hospital sources, this occupational gradient is consistent with the hypothesis that workplace exposure to antibiotic-resistant organisms contributes to colonization risk. Previous studies have similarly identified the hospital environment is a key reservoir for resistant bacteria [84].

Multivariable analysis in our study revealed significant risk factors for ESBL-PE colonization. Among hospital janitors, ESBL-PE colonization was independently associated with a lack of hand hygiene practice at home, antibiotic use within the past three months, and a history of UTI in the past three months. For non-hospital janitors, the significant risk factors were age ≥ 40 years and antibiotic use without a prescription within the past three months.

Among hospital janitors, a history of antibiotic use within the last three months was associated with more than threefold increased odds of harboring ESBL-PE (AOR: 3.54; 95% CI: 1.52, 8.23). This finding aligns with studies conducted among food handlers in Gondar, and Dilla, Ethiopia [19,20], and Gambia [67], as well as community based studies in Addis Ababa, Ethiopia [18]. This can be due to antibiotic use exerts a powerful selective pressure that eliminates susceptible gut bacteria, allowing pre-existing or newly acquired ESBL-PE to proliferate and colonize [85]. Moreover, a history UTI within the last three months was associated with a more than fourfold increased odds of harboring ESBL-PE (AOR: 4.93; 95% CI: 2.17, 11.20). However, this finding is inconsistent with the study conducted among food handlers in Gondar, Ethiopia [20], and Gambia [67], and community based studies in Turkey [86], reported that history of UTI had no significant association with the fecal carriages of ESBL-PE. Furthermore, a lack of hand hygiene practice at home was strongly associated with significantly increased odds of harboring ESBL-PE (AOR: 16.39; 95% CI: 2.14, 125.50). This finding is supported by mathematical modeling study, showing that improving hand hygiene compliance in households can reduce the probability of ESBL-PE transmission by 33–62%, highlighting the critical role of hand hygiene in interrupting the primary fecal-oral route of transmission [87].

Among non-hospital janitors who participated in this study, age ≥ 40 years was significantly associated with increased odds of ESBL-PE carriage (AOR: 6.38; 95% CI: 1.03, 39.40). This finding is consistent with a community-based study conducted in Addis Ababa, Ethiopia [18]. Additionally, antibiotic use without a prescription in the past three months was also a significant risk factor (AOR: 4.79; 95% CI: 1.72, 13.33). The discrepancy with another study among food handler in Gambia [67], which reported no significant association, may be explained by differences in regional antibiotic misuse patterns, cultural self-medication behaviors, or variations in antimicrobial access and prescribing regulations [88,89].

### Limitations of the study

The present study has some limitations. First, its cross-sectional design prevents any inference of causality and did not allow for the follow-up treatment or monitoring of carriers. Second, risk factor data were collected via questionnaire and may be subject to recall bias. Third, the majority of participants were female, which may limit the generalizability of the findings to a broader population. Fourth, wide CIs in some AORs, particularly for lack of hand hygiene at home and age ≥ 40 years, likely due to small sample size, which may lead to estimate instability. Therefore, these findings should be interpreted with caution and require confirmation in larger, adequately powered studies. Fifth, the absence of the recommended QC strain for amoxicillin-clavulanic acid may affect the reliability of results for this antibiotic, and we recommend that future studies include ATCC 35218. Finally, antibiotic resistance was determined phenotypically; confirmatory molecular detection of resistance genes was not possible due to limited resources.

## Conclusion

This study provides evidence that janitors in Ethiopia carry a significant burden of ESBL-PE and CPE, with hospital janitors exhibiting notably higher fecal carriage of ESBL-PE and CPE than non-hospital janitors, (28.6% vs. 13.7%, and 5.9% vs. 1.1%, respectively). The key predictors of ESBL-PE carriage were a lack of hand hygiene at home, antibiotic use in the past three months, and history of UTI in the past three months. For non-hospital janitors, age ≥ 40 years and antibiotic use without prescription in the past three months were significant. These findings highlight janitors, particularly these working in hospital settings, as an important reservoir for ESBL-PE and CPE.

### Recommendations

**For janitors and their families:**

- We recommend consistent practice of hand hygiene with soap and water, particularly after work, before handling food, and after using the toilet.
- Use antibiotics only with a prescription; complete the full course; do not share leftovers.
- Attend workplace or local health center training on infection prevention when available.

**For the University of Gondar (UoG):**

- Recognize janitors in hospital and non-hospital settings as an occupational risk group for carriage of AMR bacteria.
- Provide regular, practical IPC training on hand hygiene, PPE use, safe waste handling, and risks of antibiotic self-medication.
- Ensure reliable access to soap, water, hand sanitizer, and PPE at all work sites.

**For public health policy and practice:**

- Include janitors as a high-risk occupational group in national and hospital AMR surveillance programs.
- Implement IPC training for janitors in health bureaus and hospitals, focusing on hand hygiene and non-prescription antibiotic use.
- Strengthen and enforce policies prohibiting non-prescription antibiotic sales in community pharmacies.

**For future research:**

- Use whole-genome sequencing to identify resistance genes/plasmids and compare with clinical/environmental isolates.
- Conduct prospective cohort studies to establish causality between risk factors and colonization.
- Perform intervention studies assessing tailored hand hygiene and antibiotic stewardship programs.
- Include non-fermenting Gram-negative bacteria (e.g., *Acinetobacter*, *Pseudomonas*) to fully assess carbapenemase-producer burden.

## Supporting information

S1 File Participant information sheet, informed consent form and Questionnaire. (DOCX)

S2 File Culture media preparation, Gram staining, Biochemical tests, and phenotypic characteristics of Enterobacteriaceae isolates. (DOCX)

S1 Table Zone diameter disk diffusion breakpoint for Enterobacteriaceae (CLSI 2024 Guideline). (DOCX)

S2 Table Disk diffusion quality control (QC) ranges for antimicrobial agents (CLSI 2024 Guideline). (DOCX)

## Abbreviations

AMR: Antimicrobial Resistance
AOR: Adjusted Odds Ratio
AST: Antimicrobial Susceptibility Test
ATCC: American Type Culture Collection
CMHS: College of Medicine and Health Sciences
COR: Crude Odds Ratio
ESBL-PE: Extended Spectrum Beta-Lactamase -Producing Enterobacteriaceae
CPE: Carbapenemase-Producing Enterobacteriaceae
TSB: Tryptic Soy Broth
ATCC: Test American Type Culture Collection
BAA: Bioresource Accession Number
GI: Gastrointestinal
GNB: Gram-Negative Bacteria
HAIs: Hospital-Acquired Infections
HCWs: Healthcare Workers
ICU: Intensive Care Unit
OXA: Oxacillinases
IPC: Infection Prevention and Control
LMICs: Low and Middle Income Countries
MHA: Mueller-Hinton Agar
PI: Principal Investigator
PPE: Personnel Protective Equipment
QC: Quality Control
SBMLS: School of Biomedical and Laboratory Sciences
SPSS: Statistical Package for Social Science
TSB: Tryptic Soy Broth
UK: United Kingdom
UoG: University of Gondar
UoGCSH: University of Gondar Comprehensive Specialized Hospital
UTI: Urinary Tract Infection
CDC: Centers for Disease Control and Prevention
CI: Confidence Interval
CLSI: Clinical and Laboratory Standards Institute
MDR: Multidrug Resistance/Resistant
WHO: World Health Organization
