# Supplementary material for: Fecal carriage of ESBL and Carbapenemase-producing Enterobacteriaceae, and its associated factors among hospital and non-hospital janitors at the University of Gondar, Northwest Ethiopia: A comparative cross-sectional study
Source: PLoS One. 2026 Jul 31;21(7):e0355041. doi: 10.1371/journal.pone.0355041 (PMC13426960; doi:10.1371/journal.pone.0355041)
Supplement: S1 File — (DOCX) [file pone.0355041.s001.docx]

**S1_docx:** Participant information sheet, informed consent form and Questionnaire.

**English version of participant information sheet and informed consent form**

**Information sheet**

How are you? My name is ________________; I am a data and sample collector for the study that will be done by Amanuale Zayede who is studying his master’s degree in Medical Microbiology at the University of Gondar. I request you to give your attention to highlight you some information about the study.

**Study title:** fecal carriage of ESBL-PE, CPE and its associated factors among hospital and non-hospital janitors at the UoG, Northwest Ethiopia.

**Purpose of the study:** Aim to assess fecal carriage of ESBL-PE**,** CPE and its associated factors among hospital and non-hospital janitors at the UoG, Northwest Ethiopia.

**Procedure:** For this study to be successful I need your information and I am asking you to participate voluntarily in this study. If you are volunteer you are expected to understand and sign the informed consent. The sociodemographic, behavioral, occupational, hygiene and clinical information related to ESBL-PE and CPE will be filled on the questionnaire, and Stool sample will be collected for laboratory analysis.

**Risk of participation:** There is no known potential risk for participating in this study except you will spend a maximum of 15 minutes for interview and you will give small amount of stool sample.

**Compensation**: There is no special incentive you would get by participating in the study, but you will get information about your medical condition related to ESBL-PE and CPE without any price.

**Confidentiality**: We will make every effort to ensure that your information is kept as confidential as possible. For example, we will not use your name or other identifying information on study documents, stool samples or in any publications; we will replace it with an identification number. Only those taking your consent today and the principal survey investigator will be able to link your name to your survey identification number. The consent forms bearing your name and signature will be kept stored in locked cupboards. Positive results will be referred to the attending physicians for the management of the study participant’s by the principal investigator.

**Right of participants:** Participation in this study is voluntary, and you may withdraw at any time without penalty or loss of benefits. Refusal to participate will not affect your medical care.

**Contact address:** If you have any questions or concerns about this study, you can contact Amanuale Zayede (Principal investigator of this study), using phone number; +251982503000 or using Email address; [amanualezayede@gmail.com](mailto:amanualezayede@gmail.com). **Or you can contact advisors:**

Dr. Mucheye Gizachew (PhD, Associate Professor of Medical Microbiology): Phone number**;** +251910139535 or Email address; muchegiza@gmail.com

Dr. Yitayih Wondimeneh (PhD, Associate Professor of Medical Microbiology): phone number**;** +251918788124 or Email address of yitayihlab@gmail.com

Mr. Sirak Biset (MSc, Assistant Professor of Medical Microbiology): phone number**;** +251911598568 or Email address; serbis33@gmail.com

**Consent form**

After reading the information sheets or listening to the data collector, I have clearly understands the purpose of the study, the procedure, the right and I have been given chance to ask question for things that were unclear. I understand that my participation in this study is voluntary, and I may withdraw at any time without penalty or loss of benefits. I understand that there are no known potential risks associated with participating in this study, and all information collected during this study will be kept strictly confidential, and my identity will be kept anonymous. Therefore, I declare my voluntary consent to participate in this study with my Signature (initials) as indicated below.

**Participant Signature Line**

Study Participant

Name: ________________ Signature: __________ Date: _________

**Research Signature Line**

Person Obtaining Consent

Name: ________________ Signature: __________ Date: _________

**Questionnaire**

First, I would like to acknowledge you for your time and support to help me to fill this questionnaire. The aim of this questionnaire is to gather information for the study aim to assess “fecal carriage of ESBL-PE**,** CPE and its associated factors among hospital and non-hospital janitors at the University of Gondar, Northwest Ethiopia”.

The success of this research relies on you by providing timely and right information upon request. Finally, you are kindly requested to provide information honestly and responsibly.

Therefore, do you agree to participate in the study?

Yes, thank you. Signature______________. So let’s proceed to the interview

No, thank you

Please write (**√**) in front of your answer

| Questionnaire  Study code _____________________  Date of visit: __ __/__ ___/__ __ __ __ GC (day/month/year)  University of Gondar: _______________________ | | |
| --- | --- | --- |
| No. | Variable | |
| **Part-I:** **Sociodemographic characteristics** | | |
| 1 | Sex | 1. Male 2. Female |
| 2 | Age (year) | ­­­­­------ |
| 4 | Marital status | 1. Single 2. Married 3. Divorced 4. Widowed |
| 5 | Family size | ------ |
| 6 | Do you currently living with children? | 1. Yes 2. No |
| 7 | Education status | 1. No formal education 2. Primary school (1-8 grade) 3. Secondary school (9-12 grade) 4. Diploma and above |
| **Part-II: Occupational Risk Assessment** | | |
| 1 | Years of experience as a janitor? | ------- |
| 2 | Where is your working area? | 1. Hospital 2. Non-hospital |
| 3 | If Hospital, which department? | 1. Outpatient wards 2. Inpatient ward 3. Emergency room 4. Intensive care unit 5. Laboratory room 6. Operation room 7. Pediatric ward 8. Other departments |
| 4 | When is your work shift? | 1. Day 2. Night 3. Rotating shifts |
| 5 | Have you received any formal training regarding infection control in your workplace? | 1. Yes 2. No |
| 6 | Do you have access to appropriate personal protective equipment (PPE) such as masks, gloves, and gowns? | 1. Yes 2. No 3. Sometimes |
| 7 | Do you have access to handwashing facilities in work area? | 1. Always available 2. Often unavailable   3. No access |
| **Part- III: Hygiene Risk Assessment** | | |
| 1 | Do you use gloves while cleaning? | 1. Always 2. Only for dirty tasks 3. Rarely 4. Never |
| 2 | Have you exposed to surfaces contaminated with bodily fluids (blood, feces, and urine)? | 1. Frequently 2. Occasionally 3. Never |
| 3 | How often should you wash your hands to maintain proper hygiene? | 1. After every cleaning task 2. Every hour 3. Only after using restroom and before eating 4. Only when hands are visibly dirty |
| 4 | Do you clean your hands immediately after handling trash or waste materials? | 1. Yes 2. No 3. Sometimes |
| 5 | How can you wash your hands? | 1. Only with water 2. With soap and water 3. With alcohol-based sanitizer |
| 6 | What is the status of your fingernails? | 1. Trimmed 2. Non- trimmed |
| 7 | Do you have access to clean and safe toilet facilities at home? | 1. Yes 2. No |
| 8 | Do you practice hand hygiene at home (e.g., washing hands after using the toilet, before eating or before preparing food)? | 1. Yes 2. No 3. Sometimes |
| 9 | What is your source of water for drinking? | 1. Tap water 2. Hand dug well water |
| **Part IV: Behavioral risk Assessment** | | |
| 1 | Have you used antibiotics without a prescription in the last 3 months? | 1. Yes 2. No |
| 2 | If yes, for how long? | - 1. Less than 1 week 2. 1–2 weeks 3. More than 2 weeks |
| 3 | Do you drink unpasteurized milk? | 1. Yes 2. No |
| 4 | Do you consume raw meat? | 1. Yes 2. No |
|  | Do you consume uncooked vegetables? | 1. Yes 2. No |
| **Part V: Clinical Risk Assessment** | | |
| 1 | Have you used antibiotics in the last three months? | 1. Yes 2. No |
| 2 | If your answer is yes, did you appropriately used the prescribed antibiotics? | 1. Yes 2. No |
| 3 | Have you experienced gastrointestinal symptoms (e.g., diarrhea, vomiting) in the last 3 months? | 1. Yes 2. No |
| 4 | Have you had a history of urinary tract infection in the last three months? | 1. Yes 2. No |
| 5 | Have you had a history of chronic disease (e.g., diabetes, hypertension, kidney disease)? | 1. Yes 2. No |
| 6 | Have you had a history of hospital admission in the last three months? | 1. Yes 2. No |
| 7 | If your answer is yes, Have you received medical instrumentation or procedures while admitted? | 1. Yes 2. No |
| 8 | Have you been in close contact with a hospitalized person in the last three months? | 1. Yes 2. No |
