# Supplementary material for: Fecal carriage of ESBL and Carbapenemase-producing Enterobacteriaceae, and its associated factors among hospital and non-hospital janitors at the University of Gondar, Northwest Ethiopia: A comparative cross-sectional study
Source: PLoS One. 2026 Jul 31;21(7):e0355041. doi: 10.1371/journal.pone.0355041 (PMC13426960; doi:10.1371/journal.pone.0355041)
Supplement: S2 File — (DOCX) [file pone.0355041.s002.docx]

**S2_docx:** Culture media preparation, Gram staining, Biochemical tests, and phenotypic characteristics of Enterobacteriaceae isolates.

**Culture media preparation**

**MacConkey agar preparation**

MacConkey agar serves as both a selective and differential growth medium, primarily designed to isolate and distinguish non-fastidious gram-negative rods, with a focus on the Enterobacteriaceae family. Its applications include isolating gram-negative enteric bacteria and differentiating between those that ferment lactose and those that do not.

**The preparation of MacConkey agar involves several key steps.**

1. The medium was prepared from a commercial dehydrated powder according to the manufacturer's guidelines.
2. The powder was mixed with distilled water and heated to a boil until completely dissolved.
3. The solution was sterilized by autoclaving at 121°C (15 psi) for 15 minutes.
4. The sterilized medium was transferred to a Class II Biosafety Cabinet and allowed to cool to 45–50°C.
5. Once cooled and mixed, the medium was poured into sterile, flat-bottomed Petri dishes on a level surface to achieve uniform thickness (~4 mm). This required approximately 60–70 ml for larger plates (150 mm) and 25–30 ml for standard plates (100 mm).
6. The plates were left undisturbed until the agar solidified completely.
7. The prepared plates were stored at 2–8°C.
8. **Sterility Check:** 5% of each prepared batch was randomly selected, incubated at 35°C ± 2°C for 16–18 hours, and checked for any microbial growth.
9. **Performance Check:** The functionality of each new batch was verified using the *E. coli* ATCC 25922 standard reference strain.
10. Prepared plates were used within seven days unless sealed (e.g., with plastic wrap) to prevent dehydration.

**Result Interpretation:**

- Lactose-fermenting Enterobacteriaceae: Form red or pink colonies.
- Lactose non-fermenting Enterobacteriaceae: Appear as colorless, transparent colonies that do not change the medium's color (1, 2).

**Mueller-Hinton agar Preparation**

Mueller-Hinton agar is the standard medium used for antibiotic susceptibility testing of non-fastidious bacteria, primarily via the Kirby-Bauer disk diffusion method. It is non-selective and non-differential, supporting the growth of a wide range of microorganisms.

**Preparation**

The medium was prepared from a commercial dehydrated powder according to the manufacturer's instructions.

1. The mixture was heated in distilled water until boiling to ensure complete dissolution.
2. It was sterilized by autoclaving at 121°C (15 psi) for 15 minutes.
3. The sterilized medium was transferred to a Class II Biosafety Cabinet and cooled to 45–50°C.
4. After mixing, it was poured into sterile, flat-bottomed Petri dishes on a level surface to achieve a uniform depth of approximately 4 mm. This requires about 60–70 ml for 150 mm plates and 25–30 ml for 100 mm plates.
5. The plates were left undisturbed for the agar to solidify.
6. The final pH was verified to be 7.3 ± 0.1 at 25°C.
7. Prepared plates were labeled and stored at 2–8°C.
8. **Sterility Check:** 5% of each batch was incubated at 35°C ± 2°C for 16–18 hours to confirm no microbial growth.
9. **Performance Check:** Each new batch was quality-controlled using the standard strain *E. coli* ATCC 25922.
10. Plates should be used within seven days unless sealed (e.g., with plastic wrap) to prevent drying (1, 2).

**Preparation of biochemical test media**

Biochemical test media were prepared according to the following standardized procedure:

1. Media were prepared from a commercially available dehydrated powder, following the manufacturer's instructions.
2. The powder was dissolved completely in distilled water by heating it to boiling.
3. The solution was sterilized by autoclaving at 121°C (15 psi) for 15 minutes.
4. The sterile medium was transferred to a Class II Biosafety Cabinet and cooled to 45–50°C. (**Note:** For Christensen’s Urea Agar, the medium was cooled to 50–55°C before aseptically adding a sterile urea solution.)
5. Once cooled and mixed, the medium was dispensed into test tubes (4.0–5.0 ml per tube for 16-mm tubes).
6. The tubes were positioned to cool and solidify on a slant, producing a long slanted surface with a shallow butt (**Note:** For Sulphide Indole Motility medium the tube placed vertical positon).
7. The prepared slants were stored at 2–8°C.
8. Sterility was confirmed for each batch by randomly selecting and incubating 5% of the tubes at 35°C ± 2°C for 16–18 hours, ensuring no microbial growth was present (1, 2).

**Gram Staining**

Gram staining is a differential staining technique used to classify bacteria into Gram-positive and Gram-negative groups based on differences in their cell wall structure, particularly the thickness of the peptidoglycan layer (3).

**Principle**

Gram staining is based on the ability of bacterial cell walls to retain or lose the crystal violet- iodine complex during decolorization. Gram-positive bacteria have a thick peptidoglycan layer that retains the crystal violet- iodine complex after alcohol treatment and appears purple/violet. While, Gram-negative bacteria have a thin peptidoglycan layer and an outer lipid membrane. The decolorizer removes the crystal violet- iodine complex, and cells take up the counterstain, appearing pink/red.

**Materials and Reagents**

Clean glass slide, bacterial culture, inoculating loop, Bunsen burner, staining rack, wash bottle (water), compound microscope with 100× oil immersion objective, crystal violet, gram’s iodine, acetone-alcohol, and safranin.

**Procedure**

1. Prepare a thin bacterial smear on a clean slide.
2. Air-dry and heat-fix the smear.
3. Flood with crystal violet for 60 seconds; rinse.
4. Flood with Gram’s iodine for 60 seconds; rinse.
5. Decolorize with alcohol for 30 seconds; rinse.
6. Counterstain with safranin for 60 seconds; rinse.
7. Air-dry and examine under oil immersion (100×).

**Result Interpretation**

Gram-positive bacteria: purple/violate cell.

Gram-negative bacteria: Pink/red cell.

**Biochemical tests for identification of Enterobacteriaceae**

Isolated bacterial colonies were identified as members of the Enterobacteriaceae family through a series of biochemical tests. These tests assessed metabolic characteristics and enzymatic activity (1).

**Triple Sugar Iron (TSI) Test**

The Triple Sugar Iron (TSI) test is a biochemical assay used to identify bacteria based on their capacity to ferment specific sugars (glucose, lactose, and sucrose) and produce hydrogen sulfide gas. The medium contains phenol red as a pH indicator, three sugars (1% lactose, 1% sucrose, and 0.1% glucose), and sodium thiosulfate plus ferrous sulfate to detect hydrogen sulfide (H₂S) production.

**Principle**

TSI agar distinguishes bacteria by their patterns of carbohydrate fermentation and H₂S generation. Fermentation produces acid, turning the pH indicator from red/orange to yellow. Gas formation is seen as bubbles or cracks in the agar. Conversely, if the organism breaks down peptone proteins instead of sugars, alkaline byproducts are created, turning the medium a deeper red.

The lower glucose concentration (0.1%) compared to lactose and sucrose (1% each) is crucial. It ensures that organisms that ferment only glucose will initially produce acid throughout the tube. On the slanted surface exposed to air, this acid quickly oxidizes, reverting the slant to a red (alkaline) color, while the sealed butt remains yellow (acidic). Organisms that can also ferment lactose or sucrose will produce excess acid, turning both the slant and butt yellow. Furthermore, H₂S production is detected when the gas reacts with ferrous sulfate in the medium, forming a visible black precipitate of ferrous sulfide, typically in the butt of the tube (4).

**Procedure**

1. Using a sterile straight inoculation needle, pick a well-isolated bacterial colony.
2. Inoculate the TSI agar slant by first stabbing the needle deep to the bottom of the tube, then streaking the needle back and forth along the slanted surface.
3. Loosen the tube cap and incubate at 35–37°C for 18–24 hours.

**Result interpretation**:

Red / Yellow: Ferments glucose only.

Yellow / Yellow: Ferments lactose and/or sucrose.

Red / Red: Does not ferment sugars.

Blackening: Produces H₂S.

Bubbles/Cracks: Produces gas.

**Simmons Citrate Agar (Citrate utilization test)**

Simmons Citrate Agar is a diagnostic medium used to differentiate members of the Enterobacteriaceae family by determining their ability to use citrate as their only carbon source.

**Principle**The medium tests an organism's capacity to metabolize citrate for energy. Sodium citrate is provided as the sole carbon compound. Bacteria that can utilize citrate possess the enzyme citrate-permease, which transports citrate into the cell where it is converted to pyruvate for energy production. As citrate is metabolized, ammonium salts in the medium are broken down into ammonia, increasing the pH and making the environment alkaline. This pH shift changes the color of the pH indicator bromothymol blue from its original green to a bright blue at a pH above 7.6, signaling a positive result (5).

**Procedure**

1. Using a sterile straight needle, pick a well-isolated bacterial colony.
2. Inoculate a citrate agar slant by first stabbing the needle to the bottom of the tube, then streaking it along the surface of the slant.
3. Incubate the loosely capped tube at 35–37°C for 18–24 hours.

**Result Interpretation**

**Positive Result:** A color change of the medium from green to a bright blue indicates citrate utilization and an alkaline reaction.

**Negative Result:** No color change (the medium remains green) indicates the organism cannot use citrate as a carbon source.

**Motility test (SIM media)**

Motility is the ability of an organism to move independently. In bacteria, this movement is primarily achieved through flagella. It is important to determine if a bacterium is motile and to distinguish motile from non-motile strains.

**Principle**
Motility is commonly assessed using Sulphide Indole Motility media (SIM). In such media, motile bacteria can migrate away from the inoculation point, producing a diffuse, cloudy growth pattern visible to the eye. After stabbing an inoculum into the center of the tube, motile organisms will grow out from the stab line, often clouding the entire tube, while non-motile bacteria will only grow along the precise path of the inoculation (6).

**Procedure**

1. Using a sterile straight needle, pick a colony from a young (18-24 hour) agar culture.
2. Carefully stab the needle into the center of the semi-solid agar medium to a depth of 1/3 to 1/2 inch. Withdraw the needle along the exact same path to avoid spreading.
3. Incubate the tube at 35–37°C and observe daily for up to 7 days.
4. Look for a diffuse zone of growth extending from the inoculation line.

**Result Interpretation**

**Positive (Motile):** Hazy, spreading growth that makes the medium appear slightly opaque or cloudy throughout.

**Negative (Non-Motile):**Growth is strictly confined to the stab line, leaving the rest of the medium clear and transparent.

**Indole test (SIM media)**

The indole test identifies bacterial species based on their ability to produce the enzyme tryptophanase, which breaks down the amino acid tryptophan into indole and other byproducts. Following incubation, the addition of Kovac's reagent produces a red color in the presence of indole. This test is clinically useful for differentiating between certain pathogens, such as indole-negative *P. mirabilis* and indole-positive *P. vulgaris*, indole-negative *K. pneumoniae* and indole-positive *K. oxytoca*, as well as indole-negative *C. freundii* and indole-positive *C. koseri* (7).

**Procedure**

1. Inoculate a sterile tube containing tryptophan (SIM medium) with a 18-24 hour bacterial culture.
2. Incubate the tube at 37°C for 24-28 hours.
3. Add approximately 0.5 ml of Kovac's reagent to the tube.

**Result Interpretation**

**Positive:** Formation of a pink to red ring in the reagent layer shortly after adding Kovac's reagent.

**Negative:** No color change; the reagent layer remains its original yellow color.

**Lysine decarboxylase test (LDC)**

Lysine decarboxylase test (LDC) slants tests organisms for the ability to decarboxylase lysine. It is an anaerobic process which occurs in the butt of the media.

**Principle**

Lysine decarboxylase test (LDC) medium contains lysine, peptones, small amount of glucose, ferric ammonium citrate, and sodium thiosulfate. The medium has an aerobic slant and an anaerobic butt.

When glucose is fermented, the butt of the medium becomes acidic (yellow). If the organism produces lysine decarboxylase, cadaverine is formed. Cadaverine neutralizes the organic acids formed by glucose fermentation, and the butt of the medium reverts to the alkaline state (purple). If the decarboxylase is not produced, the butt remains acidic (yellow). Bromocresol purple, the pH indicator, is yellow at or below pH 5.2 and purple at or above pH 6.8 (8).

**Procedure**

1. With a straight inoculating needle, inoculate LDC by twice stabbing through the center of the medium to the bottom of the tube and then streaking the slant.
2. Cap the tube tightly and incubate at 35°-37°C in ambient air for 18 to 24 hours.
3. Examine at 18 – 24 and 40 – 48 hours for growth and color changes in tube butt and slant, and for blackening at the apex of slant.

**Result interpretation**

**Positive:** Purple slant/purple butt (alkaline)

**Negative:** Purple slant/yellow butt (acid), fermentation of glucose only.

**H_2_S production:** Black precipitate

**Gas production:** demonstrated by the presence of bubbles or cracks in the medium

**Christensen Urea Agar (Urease test)**

This test is used to differentiate organisms based on their ability to hydrolyze urea with the enzyme urease.

**Principle**

Urea is the product of decarboxylation of amino acids. Hydrolysis of urea produces ammonia and CO2. The formation of ammonia alkalinizes the medium, and the pH shift is detected by the color change of phenol red from light orange (yellow) at pH 6.8, pink at pH 8.1 (9).

**Procedure**

1. Streak the surface of a urea agar slant with a portion of a well-isolated colony.
2. Leave the cap on loosely and incubate the tube at 35°-37°C in ambient air for 48 hours to 7 days.
3. Examine for the development of a pink color for as long as 7 days.

**Result interpretation**

**Positive:** produce bright pink color.

**Negative:**  No color change (yellow color).

**Phenotypic characteristics of Enterobacteriaceae isolates based on colony morphology and biochemical tests**

**Escherichia coli** is a Gram-negative, rod-shaped, motile microorganism. When cultured on MacConkey agar, it typically forms colonies that appear red due to fermenting of lactose. Biochemically, it is characterized by a positive indole test, a negative urease test, a negative citrate utilization test, a positive LDC test, and the production of gas. It does not produce hydrogen sulfide (10).

**Klebsiella pneumoniae** is a non-motile, Gram-negative bacillus. On MacConkey agar, it forms distinctive large, mucoid, red colonies as a result of lactose fermentation. Its characteristic biochemical profile includes positive reactions for urease, citrate utilization, and LDC, along with gas production. It tests negative for indole production and hydrogen sulfide (11).

**Klebsiella oxytoca** is a non-motile, Gram-negative bacillus. On MacConkey agar, it forms red colonies due to lactose fermentation; these colonies are typically less conspicuously mucoid than those of K. pneumoniae. Its characteristic biochemical profile includes positive reactions for indole production, citrate utilization, urease activity and LDC, along with gas production. It tests negative H₂S production (12).

**Citrobacter freundii** is a motile, Gram-negative rod. On MacConkey agar, it typically forms smooth, convex, translucent to opaque grey colonies with a shiny surface and entire margin; some strains may appear mucoid or rough. It ferments lactose with gas production. Biochemically, it is characterized by being urease and H₂S positive, while testing negative for indole production and LDC (13).

**Citrobacter koseri** is a motile, Gram-negative bacillus. On MacConkey agar, it forms smooth, convex, and typically pink colonies due to lactose fermentation. Biochemically, it is characterized by positive reactions for indole production and citrate utilization, while testing variable for urease activity and H₂S production. It is a gas producer in carbohydrate fermentation tests (14).

**Enterobacter cloacae** is a motile, Gram-negative bacillus. It ferments lactose on MacConkey agar, typically resulting in pink to red colonies, and produces gas. Its biochemical profile is marked by positive citrate utilization, while it tests negative for indole production, urease activity, H₂S production, and LDC (15).

**Enterobacter aerogens** is a motile, Gram-negative bacillus. It ferments lactose on MacConkey agar, forming smooth, pink to red colonies, and is a gas producer. Its characteristic biochemical profile includes positive reactions for citrate utilization and LDC. It tests negative for indole production, urease activity, and H₂S production (16).

**Proteus species** are Gram-negative, motile bacilli. When cultivated on MacConkey agar, they produce colorless colonies due to their inability to ferment lactose. A key distinguishing biochemical feature is their strong urease activity. They are typically citrate positive, produce H₂S, and are LDC positive. Indole production is variable among species within this genus (17).

**Shigella species** are non-motile, Gram-negative bacilli. On MacConkey agar, they form smooth, colorless colonies due to their inability to ferment lactose. Biochemically, they are negative for citrate utilization, urease activity, H₂S production, and LDC. As a rule, they are non-gas producers in carbohydrate fermentation tests, with the notable exception of Shigella flexneri serotype 6. Indole production is variable and serves as a key differential characteristic among species (17).

***Serratia* species** are motile, Gram-negative bacilli. They do not ferment lactose on MacConkey agar, resulting in colorless colonies. Biochemically, they are characterized by positive citrate utilization and DNase activity. They typically test negative for indole production, urease activity, H₂S production, LDC, and are non-gas producers (17).

***Providencia rettgeri*** is a motile, Gram-negative bacillus. When cultivated on MacConkey agar, it produces small, round, convex, colorless colonies due to its inability to ferment lactose. Biochemically, it is primarily characterized by being urease and indole-positive. It tests positive for citrate utilization but negative for H₂S production and LDC, and it is a non-gas producer (18).

***Providencia stuartii*** is a motile, Gram-negative bacillus. When cultivated on MacConkey agar, it produces colorless colonies due to its inability to ferment lactose. Its defining biochemical characteristics are that it is indole- positive and urease-negative. It utilizes citrate but tests negative for H₂S production and LDC, and it is a non-gas producer (19).

**References**

1. Cheesbrough M. District laboratory practice in tropical countries, part 2: *Cambridge University Press*; 2005.

2. Rouf A, Kanojia V, Naik H, Naseer B, Qadri T. An overview of microbial cell culture. *Journal of Pharmacognosy and Phytochemistry*. 2017;6(6):1923-8.

3. Microbes Notes. Gram Stain: Principle, Reagents, Procedure, and Result Interpretation 2022 [Available from: <https://microbenotes.com/gram-stain-principle-reagents-procedure-and-result-interpretation/>.

4. Microbiology Info. The Triple Sugar Iron (TSI) Test – Principle, Procedure, Uses and Interpretation.; 2022 [Available from: <https://microbiologyinfo.com/triple-sugar-iron-tsi-test/>.

5. Microbiology Info. Simmons Citrate Agar- Composition, Principle, Uses, Preparation and Result Interpretation.; 2022 [Available from: <https://microbiologyinfo.com/simmons-citrate-agar-composition-principle-uses-preparation-and-result-interpretation/>.

6. Microbiology Info. Motility Test – Principle, Procedure, Uses and Interpretation; 2022 [Available from: <https://microbiologyinfo.com/motility-test/>.

7. Microbiology Info. Indole Test- Principle, Reagents, Procedure, Result Interpretation and Limitations.; 2022 [Available from: <https://microbiologyinfo.com/indole-test-principle-reagents-procedure-result-interpretation-and-limitations/>.

8. Microbiology Info. Lysine Iron Agar (LIA) Slants Test – Procedure, Uses and Interpretation; 2022 [Available from: <https://microbiologyinfo.com/lysine-iron-agar-slants-test/>.

9. Microbiology Info. Urease Test- Principle, Media, Procedure and Result.; 2022 [Available from: <https://microbiologyinfo.com/?s=Christensen%E2%80%99s+Urea+Agar>+.

10. Microbiology Info. Biochemical Test and Identification of E. coli 2022 [Available from: <https://microbiologyinfo.com/biochemical-test-and-identification-of-e-coli/>.

11. Microbiology Info. Biochemical Test and Identification of K. pneumoniae 2022 [Available from: <https://microbiologyinfo.com/biochemical-test-and-identification-of-klebsiella-pneumoniae/>.

12. Microbes Notes. Biochemical Test of Klebsiella oxytoca 2022 [Available from: <https://microbenotes.com/biochemical-test-of-klebsiella-oxytoca/>.

13. Microbiology Info. Biochemical Test and Identification of Citrobacter freundii 2022 [Available from: <https://microbiologyinfo.com/biochemical-test-and-identification-of-citrobacter-freundii/>.

14. Rowe B, Gross R, Allen H. Citrobacter koseri. II. Serological and biochemical examination of Citrobacter koseri strains from clinical specimens. *Epidemiology & Infection*. 1975;75(1):129-34.

15. Microbiology Info. Biochemical Test and Identification of Enterobacter cloacae 2022 [Available from: <https://microbiologyinfo.com/biochemical-test-and-identification-of-enterobacter-cloacae/>.

16. Microbes Notes. Biochemical Test of Enterobacter aerogenes 2022 [Available from: <https://microbenotes.com/biochemical-test-of-enterobacter-aerogenes/>.

17. Microbes Notes. Enterobacteriaceae: Characteristics, Identification 2025 [Available from: <https://microbenotes.com/enterobacteriaceae/#biochemical-tests-for-identification-of-common-enterobacteriaceae>.

18. Wikipedia contributors. Biochemical characteristics of Providencia rettgeri. Wikipedia, the free encyclopedia; 2025 [Available from: <https://en.wikipedia.org/wiki/Providencia_rettgeri#Biochemical_characteristics_of_Providencia_rettgeri>.

19. Uto LR, Gerriets V. Clavulanic acid. 2019.
