# Supplementary material for: Fecal carriage of ESBL and Carbapenemase-producing Enterobacteriaceae, and its associated factors among hospital and non-hospital janitors at the University of Gondar, Northwest Ethiopia: A comparative cross-sectional study
Source: PLoS One. 2026 Jul 31;21(7):e0355041. doi: 10.1371/journal.pone.0355041 (PMC13426960; doi:10.1371/journal.pone.0355041)
Supplement: S1 Table — (DOCX) [file pone.0355041.s003.docx]

| **Antimicrobial**  **Agents** | **Disk content** | **Read times, hours** | **Interpretive categories and zone diameter breakpoints, nearest whole number in mm** | | |
| --- | --- | --- | --- | --- | --- |
|  |  |  | **S** | **I** | **R** |
| **Beta-lactam combination agents** | | | | | |
| Amoxicillin/clavulanic acid | 20/10 µg | 16-18 | ≥18 | 14-17 | ≤13 |
| **Cephalosporins (II, and III)** | | | | | |
| Cefoxitin/CXT | 30 µg | 16-18 | ≥18 | 15-17 | ≤14 |
| Cefotaxime/CTX | 30 µg | 16-18 | ≥26 | 23-25 | ≤22 |
| Ceftriaxone/CRO | 30 µg | 16-18 | ≥23 | 20-22 | ≤19 |
| Ceftazidime/CAZ | 30 µg | 16-18 | ≥21 | 18-20 | ≤17 |
| **Carbapenem** | | | | | |
| Meropenem | 10 µg | 16-18 | ≥22 | 19-21 | ≤18 |
| Imipenem | 10 µg | 16-18 | ≥22 | 20-22 | ≤19 |
| **Aminoglycosides** | | | | | |
| Gentamicin | 10 µg | 16-18 | ≥18 | 15-17 | ≤14 |
| Amikacin | 30 µg | 16-18 | ≥20 | 17-19 | ≤16 |
| **Tetracyclines** | | | | | |
| Tetracycline | 30 µg | 16-18 | ≥15 | 12-14 | ≤11 |
| **Fluoroquinolones for Enterobacteriaceae except for *Salmonella* spp**. | | | | | |
| Ciprofloxacin | 5 µg | 16-18 | ≥21 | 18-20 | ≤17 |
| **Folate pathway Antagonists** | | | | | |
| Trimethoprim-sulfamethoxazole | 1.25/23.75 µg | 16-18 | ≥16 | 11-15 | ≤10 |
| **Phenicols** | | | | | |
| Chloramphenicol | 30 µg | 16-18 | ≥18 | 13-17 | ≤12 |

**S1 Table:** Zone diameter disk diffusion breakpoint for Enterobacteriaceae (CLSI 2024 Guideline).
