# Supplementary material for: Fecal carriage of ESBL and Carbapenemase-producing Enterobacteriaceae, and its associated factors among hospital and non-hospital janitors at the University of Gondar, Northwest Ethiopia: A comparative cross-sectional study
Source: PLoS One. 2026 Jul 31;21(7):e0355041. doi: 10.1371/journal.pone.0355041 (PMC13426960; doi:10.1371/journal.pone.0355041)
Supplement: S2 Table — (DOCX) [file pone.0355041.s004.docx]

| **Antimicrobial agents** | **Disk content in µg** | **Disk diffusion QC ranges in mm** |
| --- | --- | --- |
|  |  | *E. coli* ATCC 25922 |
| Cefoxitin | 30 | 23-29 |
| Cefotaxime | 30 | 29-35 |
| Ceftazidime | 30 | 25-32 |
| Ceftriaxone | 30 | 29-35 |
| Chloramphenicol | 30 | 21-27 |
| Ciprofloxacin | 5 | 29-38 |
| Gentamicin | 10 | 19-26 |
| Amikacin | 30 | 19-26 |
| Imipenem | 10 | 26-32 |
| Meropenem | 10 | 28-35 |
| Tetracycline | 30 | 18-25 |
| Trimethoprim-sulfamethoxazole | 1.25/23.75 | 23-29 |
| Amoxicillin-clavulanic acid (2:1) | 20/10 | 18-24 |

**S2 Table:** Disk diffusion quality control (QC) ranges for antimicrobial agents (CLSI 2024 Guideline).
